# Identification of Potential Immune-Related circRNA–miRNA–mRNA Regulatory Network in Intestine of *Paralichthys olivaceus* During *Edwardsiella tarda* Infection

**DOI:** 10.3389/fgene.2019.00731

**Published:** 2019-08-14

**Authors:** Yunji Xiu, Guangpeng Jiang, Shun Zhou, Jing Diao, Hongjun Liu, Baofeng Su, Chao Li

**Affiliations:** ^1^School of Marine Science and Engineering, Qingdao Agricultural University, Qingdao, China; ^2^Shandong Key Laboratory of Disease Control in Mariculture, Marine Biology Institute of Shandong Province, Qingdao, China; ^3^School of Fisheries, Aquaculture and Aquatic Sciences, Auburn University, Auburn, AL, United Statess

**Keywords:** circRNA, miRNA, mRNA, circRNA–miRNA–mRNA network, immune response, *Paralichthys olivaceus*, *Edwardsiella tarda*

## Abstract

Olive flounder (*Paralichthys olivaceus*) is an important economical flatfish in Japan, Korea, and China, but its production has been greatly threatened by disease outbreaks. In this research, we aimed to explore the immune responsive mechanism of *P. olivaceus* against *Edwardsiella tarda* infection by profiling the expression of circRNA, miRNA, and mRNA by RNA-seq and constructing a regulatory circular circRNA–miRNA–mRNA network. Illumina sequencing of samples from normal control (H0), 2 h (H2), 8 h (H8), and 12 h (H12) post-challenge was conducted. Differentially expressed (DE) circRNA (DE–circRNAs), miRNAs (DE–miRNAs), and mRNAs [differential expression genes (DEGs)] between challenge and control groups were identified, resulting in a total of 62 DE–circRNAs, 39 DE–miRNAs, and 3,011 DEGs. Based on the differentially expressed gene results, miRNA target interactions (circRNA–miRNA pairs and miRNA–mRNA pairs) were predicted by MiRanda software. Once these paired were combined, a preliminary circRNA–miRNA–mRNA network was generated with 198 circRNA–miRNA edges and 3,873 miRNA–mRNA edges, including 44 DE–circRNAs, 32 DE–miRNAs, and 1,774 DEGs. Kyoto Encyclopedia of Genes and Genomes (KEGG) analysis was performed to evaluate the function of the DEGs in this network, and we focused and identified two important intestinal immune pathways (herpes simplex infection and intestinal immune network for IgA production) that showed statistical significance between the challenge and control groups. Furthermore, three critical DEGs (nectin2, MHC II α-chain, and MHC II β-chain) were identified, mapped into the preliminary circRNA–miRNA–mRNA network, and new circRNA–miRNA–mRNA regulatory networks were constructed. In conclusion, we, for the first time, identified circRNA–miRNA–mRNA network from *P. olivaceus* in the pathogenesis of *E. tarda* and provided valuable resources for further analyses of the molecular mechanisms and signaling networks.

## Introduction

Circular RNAs (circRNAs), identified from RNA viruses in the 1970s (Sanger et al., 1976), were initially treated as viral genomes or by-products of rare mis-splicing, and thus, they have long been thought to be nonfunctional (Capel et al., 1993). CircRNAs are generated during the process of back-splicing and could be grouped into four categories: circular exonic RNAs (ecircRNAs), circular intronic RNAs (ciRNAs), exon–intron circRNAs (eiciRNAs), and intergenic circRNAs (Qu et al., 2015; Sonja and Sabine, 2015; Li et al., 2017a). During back-splicing, a downstream 5′ splice donor is joined with an upstream 3′ splice acceptor, and the resulting RNA circle is ligated by a 3′–5′ phosphodiester bond at the junction site (Lasda and Parker, 2014; Chen, 2016; Wilusz, 2018). Back-splicing is catalyzed by the canonical spliceosome machinery and modulated by both intronic complementary sequences and RNA binding proteins (Li et al., 2018b). Recent advancements to the high-throughput sequencing technology have benefited large amounts of circRNAs identified in succession from many organisms, such as plants, animals, human beings, fungi, and protists (Li et al., 2018b). Emerging researches suggest that some circRNAs are critical in many physiological and pathological conditions (Li et al., 2018b). For example, expression profiles and knockout experiments proved that circRNAs have been implicated in neuronal function (Rybak-Wolf et al., 2015; Piwecka and Glazar, 2017) and testes development (Capel et al., 1993; Hansen et al., 2013). Besides, more and more circRNAs have been found to be associated with human diseases, such as cancers (Conn et al., 2015; Guarnerio et al., 2016), Alzheimer’s disease (Lukiw, 2013), neuronal diseases (Errichelli et al., 2017), and others. In addition, the most recent progresses reveal that some circRNAs are also involved in innate immune responses (Chen et al., 2017b; Li et al., 2017b; Wang et al., 2017). Collectively, considerable evidences proved that circRNAs are not simply accidental by-products but represent an essential part of non-coding RNA families.

Although relevant research on circRNAs is still in its infancy, it is becoming apparent that circRNAs play their regulatory roles through distinct mechanisms. Initially, circRNAs function as miRNA sponges through abundant binding sites for microRNAs and then modulate the activity of miRNAs on their target genes (Hyeon Ho et al., 2009). Remarkably, some circRNAs are strongly associated with cancer progression through competing with miRNAs to influence the expression of target genes that are involved in biological processes, for example, tumor cell proliferation, apoptosis, invasion, and migration (Zhong et al., 2018). Apart from acting as miRNA sponges, circRNAs play multiple functions through affecting splicing of their linear mRNA counterparts, regulating transcription of their parental genes, influencing splicing of their linear cognates, interacting with associated proteins, protein-coding genes, and generating pseudogenes (Li et al., 2018b).

Olive flounder (*P. olivaceus*) is an important economical flatfish that has been widely cultured in Japan, Korea, and China. The production of *P. olivaceus* has been greatly threatened by disease outbreaks, including bacteria, virus, and parasites (Kim et al., 2010). *Edwardsiella tarda*, associated with hemorrhagic septicemia of freshwater and marine fish, could also result in extensive economic losses to aquaculture industry of *P. olivaceus* (Mohanty and Sahoo, 2007; Xu and Zhang, 2014). It was reported that *E. tarda* is an important zoonotic and intestinal pathogen, and the intestine was likely the main route of entry to host (Li et al., 2012; Wang et al., 2012). Therefore, besides serving as the prime site for absorption of nutrients, intestine represents one of the first-line defense systems (Lauriano et al., 2019). It has been confirmed that intestinal hypo-immunity of fish favors *E. tarda* infection (Liu et al., 2014). Teleost fish possess a diffuse mucosa-associated immune system in the intestine where B cells act as one of the main responders (Parra et al., 2016). Moreover, IgT^+^ B cells represent the predominant mucosal B-cell subset, and the accumulation of IgT^+^ B cells has been detected in trout intestine after infection (Yu and Kong, 2018). Immunoglobulins produced by these B cells constitute a critical line of defense, which prevents the entrance of pathogens and commensal bacteria into the epithelium (Parra et al., 2016).

It is vitally necessary to understand and apply their immune mechanism against pathogen infection. Over the years, massive efforts have been conducted in exploring the immune mechanism of *P. olivaceus* at a molecular level (Hwang et al., 2018; Ma et al., 2018; Wu et al., 2018), among which a few researches have been conducted in non-coding RNA (Zhang et al., 2014). However, there has been no report about the important roles of circRNAs during the immune process of *P. olivaceus*. In fish, few circRNA researches have been published on teleosts, including half-smooth tongue sole (*Cynoglossus semilaevis*) (Li et al., 2018a), large yellow croaker (*Larimichthys crocea*) (Xu et al., 2017), zebrafish (*Danio rerio*) (Shen et al., 2017), coelacanth (Anne et al., 2014) and grass carp (*Ctenopharyngodon idella*) (He et al., 2017). Interestingly, the most recent progresses reveal that circRNAs also take part in immune regulation and viral infection (Wang et al., 2017). It has been identified that the *in vitro* synthesized circRNAs would activate RIG-I-mediated innate immune responses, which will provide protection against viral infection (Chen et al., 2017b). Besides, the immune factors NF90/NF110 modulate circRNA biosynthesis and suppress viral infection by interacting with viral mRNAs (Li et al., 2017b). Moreover, it has been speculated that a circRNA–miRNA–mRNA network may be present in grass carp reovirus (GCRV)-infected grass carp, which provides new insights into the immune mechanism underlying grass carp against GCRV (He et al., 2017). However, there are still several intriguing questions that remained to be clarified: 1) Are circRNAs involved in antibacterial immune responses? 2) How do circRNAs contribute to antibacterial immune responses?

In this study, we examined interaction of circRNAs, miRNAs, and mRNAs of *P. olivaceus* in the pathogenesis of *Edwardsiella tarda* by high-throughput sequencing. We screened and identified differentially expressed circRNAs, miRNAs, and mRNAs; predicted the potential circRNA–miRNA–mRNA network; analyzed their significant enrichment pathways; and emphasized their implications in antibacterial immunity for the first time.

## Materials and Methods

### The Experimental Fish and Ethical Statement

Healthy olive flounders were obtained from Huanghai Aquaculture Company (Haiyang, Shandong, China). The fish were acclimatized in a recirculating water system (temperature 20 ± 1°C) for 1 week before processing, during which they were fed twice a day with commercial diet. In order to make sure that all the experimental fish were healthy, the olive flounders were monitored every day; after 1-week acclimation, they were randomly sampled for bacteriological examination. This study was carried out in accordance with the recommendations in the Guide for the Care and Use of Laboratory Animals of the National Institutes of Health, Qingdao Agricultural University. The protocol was approved by the Committee on the Ethics of Animal Experiments of Qingdao Agricultural University IACUC (Institutional Animal Care and Use Committee).

### Bacteria Challenge and Sample Collection

The bacterial of *E. tarda* were isolated from diseased olive flounders and kept by our laboratory. Before the challenge experiment, *E. tarda* were incubated in Luria broth (LB) medium at 28°C to mid-logarithmic stage. The concentration was determined by colony-forming unit (CFU) method. Overall, this experiment included four time points, and each time point contained three biological replicates (three fish for each biological replicate). Before *E. tarda* infection, nine fish were immersed in sterilized media, and their posterior intestines were sampled as the normal control group. The samples were designated as H0 (H0_1, H0_2, and H0_3). During challenge, the experimental groups were immersed in the bacteria solution with a final concentration of 6 × 10^7^ CFU/ml for 2 h and then returned to the circulating water system. At 2, 8, and 12 h post-treatment, nine fish from each time point were collected, and their posterior intestines were collected for sequencing. The samples were designated as H2 (H2_1, H2_2, and H2_3), H8 (H8_1, H8_2, and H8_3), and H12 (H12_1, H12_2, and H12_3).

### Histopathological Analysis

To observe histopathological changes of intestine in the *E. tarda* infected *P. olivaceus*, we took posterior intestines from nine fish at each time point to make pathological sections. Tissue samples were fixed in 4% paraformaldehyde in phosphate-buffered saline (PBS) and then further processed through the following steps: dehydrated in graded ethanol, cleared in xylene, embedded in paraffin, cut into 5-mm sections, and stained with hematoxylin and eosin (H&E) for examination by light microscopy (Licata et al., 2018). The histological measurements for the structures, height of mucosal folds, thickness of lamina propria, inner circular muscular layer, and outer longitudinal muscle were measured and analyzed. The mean ± standard error of mean (SEM) of each structure was compared among all of samples using the analysis of variance with Tukey LSD (SAS 9.4) at the significance level *p* < 0.05.

### RNA Isolation, Library Construction, and Sequencing

Total RNA from samples was extracted by using the TRIzol reagent (Invitrogen, USA). The purity of total RNA was checked by using the NanoPhotometer^®^ spectrophotometer, its concentration was checked by using Qubit^®^ RNA Assay Kit in Qubit^®^ 2.0 Fluorometer, and its integrity was checked by Bioanalyzer 2100 system.

At different time points before (H0) and after (H2, H8, and H12) *E. tarda* infection, intestine tissues from nine olive flounders were respectively collected and used for circRNA sequencing. Three replicate samples were processed for each time point, and a total of 12 libraries were sequenced. For constructing the library of circRNAs or mRNAs, 5 μg of RNA for each sample was prepared. Then, Epicentre Ribo-Zero™ rRNA Removal Kit (Epicentre, USA) was used to remove rRNA, and ethanol precipitation was applied to clean up rRNA-free residue. Subsequently, the linear RNA was digested with 3 U of RNase R (Epicentre, USA) per microgram of RNA for mRNA library and without RNase R treatment for circRNAs, which was the only difference between library construction of circRNAs and mRNAs. The sequencing libraries were generated by using NEBNext^®^ Ultra™ Directional RNA Library Prep Kit for Illumina^®^ (NEB, USA) according to manufacturer’s protocol. For circRNA, mRNA, and miRNAs, the library construction and sequencing were operated by Novogene Corporation (China) in the same way as previously reported (Lu et al., 2016). In consideration that the Ribo-Zero library contained both mRNA and lncRNA at the same time, but we were not interested in lncRNA in this research, we set up a series of strict screening conditions to identify and remove lncRNA according to their structural characteristics and functional characteristics. The screening process of lncRNA is shown as follows: 1) Select transcripts with exon number ≥2. 2) Select transcripts with length > 200 bp. 3) Screen transcripts that overlap with the annotated exon area. 4) Cuffquant was used to calculate the expression of each transcript, and the transcripts with fragments per kilobase of transcript per million mapped reads (FPKM) ≥0.5 were selected. (5) CNCI (coding–non-coding index) (v2), CPC (Coding Potential Calculator) (0.9-r2), Pfam Scan (v1.3), and PhyloCSF (phylogenetic codon substitution frequency) (v20121028) were used to predict the coding potential of transcripts, and the intersected transcripts without coding potential from these four software analysis were selected as the lncRNA.

### Data Analysis

Raw data (raw reads) were firstly processed through in-house perl scripts (for circRNAs and mRNAs) or customperl and python scripts (for miRNAs). More concretely, reads containing adapter, ploy-N, and low-quality reads from raw data were removed. Then, Q20, Q30, and GC contents of the clean data were calculated. All the downstream analyses were based on the clean data.

Reference genome and gene model annotation files were downloaded from genome website directly (ftp://ftp.ncbi.nlm.nih.gov/genomes/all/GCF/001/970/005/GCF_001970005.1_Flounder_ref_guided_V1.0/). Index of the reference genome was built using bowtie2 (v2.2.8), and clean reads were aligned to the reference genome using Bowtie or HISAT2 (Langmead and Pop, 2009; Langmead and Salzberg, 2012).

The circRNAs were detected and identified using find_circ (Memczak et al., 2013) and CIRI2 (Gao et al., 2018). The workflow of find_circ was as follows: 1) The reads that aligned contiguously to the genome were filtered out, and spliced reads were retained; 2) the terminal parts of each candidate read were mapped to the genome to find unique anchor positions; 3) candidate circRNAs were confirmed when their 3′ end of anchor sequence aligned to the upstream of 5′ end of anchor sequence, and the inferred breakpoint was flanked by GU/AG splice signals. The circos figures were constructed by using Circos software. Mapped small RNA tags were used to search for known miRNA. MiRBase20.0 was used as reference; modified software mirdeep2 (Friedländer et al., 2012) and srna-tools-cli were used to obtain the potential miRNA and draw the secondary structures. Custom scripts were used to obtain the miRNA counts as well as base bias on the first position of identified miRNA with certain length and on each position of all identified miRNA, respectively (Jian et al., 2018). The characteristics of hairpin structure of miRNA precursor can be used to predict novel miRNA. The available software miREvo (Wen, 2012) and mirdeep2 (Friedländer et al., 2012) were integrated to predict novel miRNA through exploring the secondary structure, the Dicer cleavage site, and the minimum free energy of the small RNA tags unannotated in the former steps. Meanwhile, custom scripts were used to obtain the identified miRNA counts as well as base bias on the first position with certain length and on each position of all identified miRNA, respectively (Fan et al., 2018). For transcriptome assembly, the mapped reads of each sample were assembled by StringTie (v1.3.1) (Pertea et al., 2016) in a reference-based approach. StringTie uses a novel network flow algorithm as well as an optional *de novo* assembly step to assemble and quantitate full-length transcripts representing multiple splice variants for each gene locus.

### Differential Expression Analysis, Enrichment Analysis, and circRNA–miRNA–mRNA Network Analysis

Differential expression analysis between two groups was performed using the DESeq R package (1.8.3). The *p*-value was adjusted using the Benjamini and Hochberg method. Corrected *p*-value of 0.05 was set as the threshold for significantly differential expression by default.

Gene ontology (GO) and Kyoto Encyclopedia of Genes and Genomes (KEGG) enrichment analyses were used on significantly differential expressed genes, including host genes of differentially expressed circRNAs and the target gene candidates of differentially expressed miRNAs. Gene ontology (GO) enrichment analysis was implemented by the GOseq R package, in which gene length bias was corrected (Young et al., 2010). GO terms with corrected *p*-value of less than 0.05 were considered significantly enriched by differentially expressed genes. KEGG is a database resource for understanding high-level functions and utilities of the biological system (Kanehisa et al., 2008), such as the cell, the organism, and the ecosystem, from molecular-level information, especially large-scale molecular datasets generated by genome sequencing and other high-throughput experimental technologies (http://www.genome.jp/kegg/). We used KOBAS software to test their statistical enrichment in KEGG pathways (Mao et al., 2005).

The circRNA–miRNA–mRNA network was developed based on possible functional relationships between DE–circRNAs, DE–miRNAs, and differential expression genes (DEGs). Firstly, the target circRNAs of DE–miRNAs were predicted by scanning for conserved miRNA target sites with MiRanda (Enright et al., 2003); then the interactions between target circRNAs and DE–circRNAs were identified; and finally circRNA–miRNA regulation network was constructed. Secondly, the target mRNAs of DE–miRNAs were predicted by scanning for conserved miRNA target sites with MiRanda; then the interactions between target mRNAs and DEGs were identified; and finally miRNA–mRNA regulation network was constructed. At last, circRNA–miRNA–mRNA network was generated using a combination of circRNA–miRNA network and miRNA–mRNA network with Cytoscape 3.6.1 software (Su et al., 2014), and only the network follows the expression trend of “up–down–up” or “down–up–down” was selected for further research. In conclusion, the construction of circRNA–miRNA–mRNA network followed the following principles: circRNAs served as bait, microRNAs served as core, and RNA served as target.

### Confirmation of the Expression Level of circRNAs, miRNAs, and mRNAs

To validate the reliability of the data obtained from Illumina sequencing, real-time quantitative reverse transcription polymerase chain reaction (qRT-PCR) and Sanger sequencing were conducted. To confirm the expression pattern of differentially expressed circRNAs, six circRNAs were randomly selected for qRT-PCR and Sanger sequencing. Primer Premier 5 software was used to design their divergent primers. In general, the divergent primers were designed to span the circRNA backsplice junction, and a fragment of 80- to 150-bp length was expected. Total RNA was extracted, digested using RNase-Free DNase (Promega), and then purified. A total of 1 μg of purified RNA was used to prepare first-strand cDNA by using a random 6 mers primers and the PrimeScript 1^st^ strand cDNA Synthesis Kit (Takara, Japan). qRT-PCR was carried out on a CFX 96 real-time PCR system (Bio-Rad, Hercules, CA, USA). Each RT-qPCR mixture contained 4.2 μl of ddH_2_O, 5 μl of 2× SYBR Green master mix (Aidlab), 0.4 μl of template, and 0.2 μl each of forward and reverse primers. The EF1α gene was used as an internal control. For each sample, three replicates were included. The program for qRT-PCR was as follows: 95°C for 3 min, followed by 40 cycles of 95°C for 10 s and 60°C for 34 s. Relative expression level was calculated using the Pfaffl method (Pfaffl, 2001). Data are shown as means ± SE of three replicates. To further prove that the amplified products were the template with circRNA rather than liner counterparts, products of qRT-PCR were subjected to pEASY-T1 cloning vector (TransGen, Beijing, China) and then sequenced by Tsingke Company (Tsingke, Qingdao, China).

Meanwhile, to further confirm the expression level of miRNAs and mRNAs, 8 miRNAs and 10 mRNAs were randomly selected for qRT-PCR experiments. For miRNAs analysis, small RNA (<200 nt) was harvested using the miRcute miRNA isolation kit (Tiangen Biotech). The amplification reactions were carried out using the miRcute miRNA qPCR detection kit (Tiangen Biotech) with the following conditions: 95°C for 15 min, 40 cycles of two steps (95°C for 5 s and 60°C for 30 s) within triplicate wells of each sample. The relative expression level of miRNA was normalized by 5S rRNA expression. For mRNAs analysis, reaction mixtures and the program for qRT-PCR were the same as circRNAs, and the EF1α gene was again used as an internal control for the normalization of gene expression.

## Results

### Histopathological Description and Cytokine Expression Analysis

**Figure 1:**
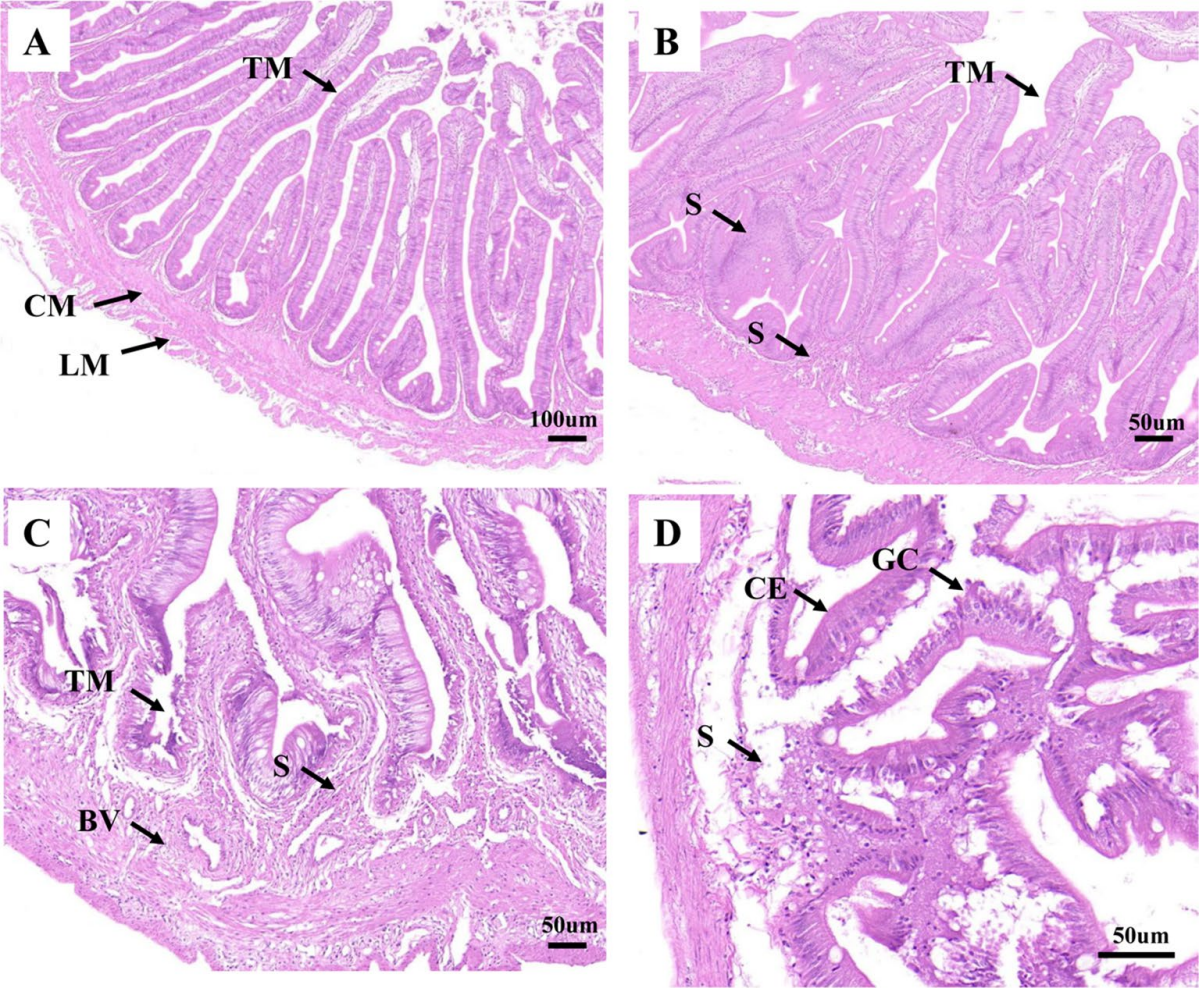
Analysis of histopathological data. **(A)**, **(B)**, **(C)**, and **(D)** represent the intestines from H0, H2, H8, and H12 time point, respectively. BV, blood vessel; CE, columnar epithelial cell; CM, circular muscularis; GC, goblet cell; LM, longitudinal muscularis; S, submucosa; TM, tunica mucosa. The scale bar in **(A)** is 100 µm. The scale bar in **(B)**, **(C)**, and **(D)** is 50 µm.

As shown in **Figure 1A**, the healthy intestine sample contained tunica mucosa, submucosa, mucosal folds, circular muscular layer, and longitudinal muscular layer. The mucosal folds were lined with abundant of columnar epithelial cells and goblet cells. And all the cells appeared to be interrelated and uniformly arranged. For H2, in early infection, hyperplasia of intestinal mucosa was observed. Meanwhile, the blood vessel and the lymph vessel enlarged, which were associated with the increasing thickness of lamina propria (**Supplementary Table 1**). However, the intestine structure was still integrated, the height of the mucosal folds decreased (**Supplementary Table 1**), and most of the mucosal folds did not show significant lesions. The columnar epithelial cells and goblet cells were also lined tightly (**Figure 1B**). For H8, cellular swelling and hydropic change were observed in intestinal epithelium, some intestinal epithelial cells were shedding, and some became disrupted. Inflammatory cells were infiltrated in connective tissue. Simultaneously, cells in the blood vessel and the lymph vessel became quantitatively more and more distinct. The intestine structure was injured severely (**Figure 1C**). For H12, the infection led to destructive damage to the intestine structures. The mucosal folds suffered from further damage; fragmentation were observed. The blood vessel was also severely damaged, and only a few blood vessels could be observed. Necrosis was seen in the mucosa, submucosa, and muscle layers of intestinal wall (**Figure 1D**). The lamina propria was significantly thicker at 12 h post-challenge (**Supplementary Table 1**; **Figure 1D**).

**Figure 2:**
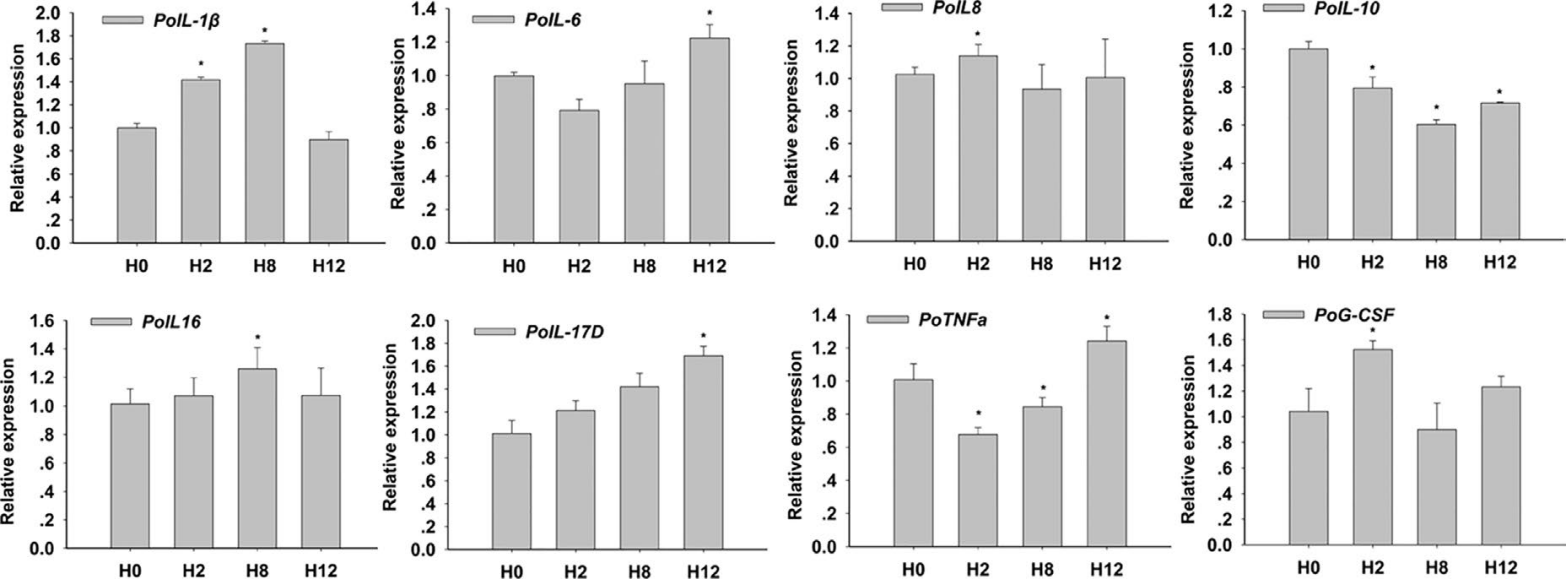
Relative expression of interleukin 1β (IL-1β), IL-6, IL-8, IL-10, IL-16, IL-17D, tumor necrosis factor α (TNF-α), and granulocyte colony-stimulating factor (G-CSF) in posterior intestines of *E. tarda*-infected *P. olivaceus*. The data present the mean ± standard error (SE) derived from triplicate experiments. * represents the values that are significantly different (*p* < 0.05) from H0 time point.

In order to clarify the biological importance for time course, the qRT-PCR check for expression of key cytokines for enteritis was conducted. The results showed that *E. tarda* could 1) up-regulate mRNA levels of intestinal pro-inflammatory cytokines interleukin *1β* (*IL-1β*), *IL-6*, *IL-8*, *IL-16*, and *IL-17D*, tumor necrosis factor α (*TNF-α*), and granulocyte colony-stimulating factor (*G-CSF*); and 2) down-regulate the mRNA levels of anti-inflammatory cytokines *IL-10* (**Figure 2**). The primers of qRT-PCR are shown in **Supplementary Table 2**.

### Overview of circRNA Sequencing Data

As shown in **Table 1**, raw reads, clean reads, clean bases, and Q20, Q30, and GC (guanine and cytosine) contents for each library were identified. All libraries gave a good quality base value ≥ 12.25 Gb, Q20 ≥ 96.99%, Q30 ≥ 92.32%, and an error rate ≤ 0.02. Therefore, all libraries proved to be suitable for further study. These data were deposited in NCBI database with the BioProject number of PRJNA511138.

**Table 1:** Information list of circRNA sequencing data.

Sample	Duplicates	Raw reads	Clean reads	Clean bases (Gb)	Error rate	Q20	Q30	GC content
H0	H0_1	91001552	85570680	12.84G	0.02	97.16	92.72	47.40
	H0_2	98086488	92224408	13.83G	0.02	97.18	92.72	48.90
	H0_3	116471068	109413634	16.41G	0.02	96.99	92.32	48.85
H2	H2_1	133971546	125928236	18.89G	0.02	97.10	92.55	49.09
	H2_2	91713816	86756860	13.01G	0.02	97.19	92.72	49.33
	H2_3	83268176	81661360	12.25G	0.02	97.29	92.87	49.59
H8	H8_1	84134060	82485038	12.37G	0.02	97.33	92.97	49.47
	H8_2	89841602	88149192	13.22G	0.02	97.33	92.98	49.26
	H8_3	85281542	83698290	12.55G	0.01	97.39	93.10	49.37
H12	H12_1	123787190	121500194	18.23G	0.01	97.39	93.09	49.28
	H12_2	99739544	97782904	14.67G	0.02	97.33	92.96	49.00
	H12_3	112061808	109856848	16.48G	0.01	97.37	93.09	47.94

**Figure 3:**
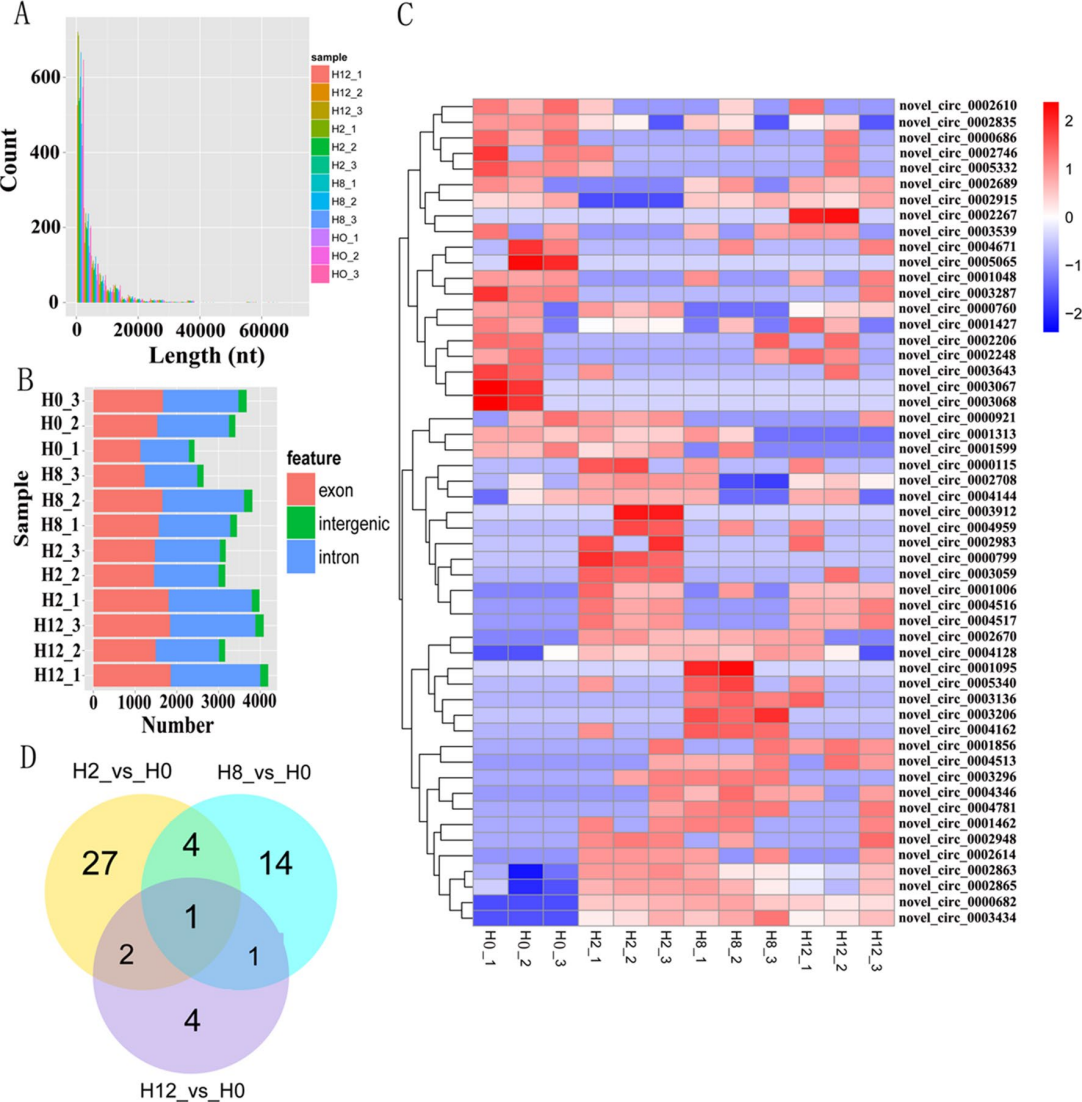
Analysis of circRNA sequencing data. **(A)** Size distribution of unique circRNA sequences from H0, H2, H8, and H12 libraries. **(B)** The number of circRNAs arising from different genomic locus (exon, intronic, and intergenic). **(C)** Heatmap was used to assess the expression of DE–circRNAs. Red and blue denote high and low expression, respectively. Each DE–circRNA is represented by a single row of colored boxes, and each sample is represented by a single column. **(D)** Pairwise comparisons of differentially expressed circRNAs between two libraries. The Venn diagrams display the distribution of the 53 unique DE–circRNAs between H2, H8, H12, and H0, respectively.

Clean reads from the 12 libraries were used to identify circRNAs. After a series of selection, 5,478 novel circRNAs were obtained and termed from novel_circ_0000001 to novel_circ_0005478 (**Supplementary Table 3**). There was no single circRNA reported in olive flounders previously, so all the identified circRNAs were novel. A size distribution analysis revealed that the length of circRNAs ranged from 150 to 71,793 bp, but most (74.82%) were ≤5,000 bp (**Figure 3A**). Most of the circRNAs were exonic and intronic (**Figure 3B**).

To identify circRNAs that potentially participated in *E. tarda* infection, their expression profiles were examined at 0, 2, 8, and 12 h post-infection. As shown in **Supplementary Table 4**, a total of 34, 20, and 8 differentially expressed circRNAs (DE–circRNAs) were observed at 2, 8, and 12 h relative to 0-h control, respectively. A heatmap showing 62 DE–circRNAs (**Figure 3C**) and a Venn diagram revealing one circRNA (novel_circ_0005065) were differentially expressed at all three comparisons (**Figure 3D**).

### Overview of miRNA Sequencing Data

**Figure 4:**
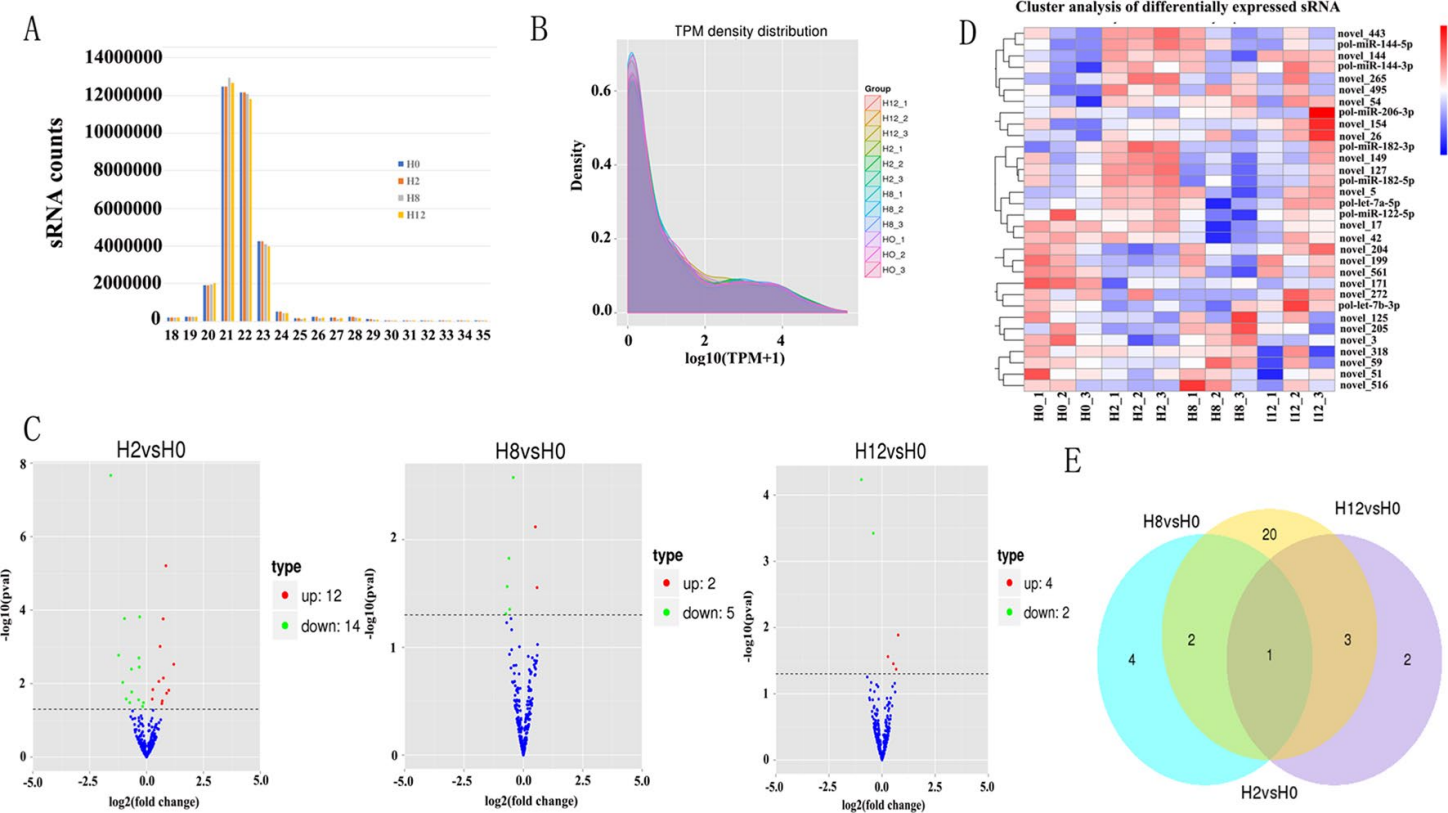
Analysis of miRNA sequencing data. **(A)** Size distribution of sRNAs from H0, H2, H8, and H12 libraries. **(B)** TPM (number of transcripts per million clean tags) density distribution of sRNAs from 12 samples. **(C)** Volcano plots for DE–miRNAs between H2, H8, H12, and H0, respectively. **(D)** Heatmap is used to assess the expression of DE–miRNAs. Red and blue denote high and low expression, respectively. Each DE–miRNA is represented by a single row of colored boxes, and each sample is represented by a single column. **(E)** Venn diagrams display the distribution of the 32 unique DE–miRNAs between H2, H8, H12, and H0, respectively.

Meanwhile, to study the miRNA profile of *P. olivaceus* after *E. tarda* infection, four sRNA libraries (i.e., H0, H2, H8, and H12) were also constructed and sequenced. Altogether, 33,957,978, 33,067,562, 34,149,754, and 33,590,933 raw reads were acquired from H0, H2, H8, and H12, respectively. These data were deposited in NCBI database with the BioProject number of PRJNA510916. After the low-quality reads, adaptor sequences, and reads with sequences < 1 or >35 nt were filtered, all sRNAs were obtained (**Table 2**). As shown in **Figure 4A**, the majority of the sRNAs from the four libraries ranged from 20 to 23 nt, and the peak distribution was for sequences that were 21 nt long. Furthermore, the sRNAs of all samples were compared with their reference genomes. The results showed that most of sRNAs (>89%) were able to be mapped onto the olive flounder genome (**Table 2**). After Rfam and genome databases were searched, other non-coding RNAs (rRNA, tRNA, snRNA, and snoRNA) (**Supplementary Table 5**) and repeat sequences (**Supplementary Table 6**) were annotated.

**Table 2:** Information list of miRNA sequencing data.

Sample	Total reads	Low quality	Clean reads	Total reads of sRNA	Uniq reads	Mapped sRNA
H0_1	11539149	130837	11275849	11127305	461189	10064134
H0_2	11409634	148506	11132958	10979861	296376	10091473
H0_3	11009195	150564	10736651	10634330	314366	9627697
H2_1	10887228	170411	10586259	10400419	387640	9299557
H2_2	11347535	141431	11089712	10926718	410080	9844333
H2_3	10832799	151584	10563175	10446171	399707	9364900
H8_1	11320500	172940	11023361	10787063	412589	9664232
H8_2	11221353	64487	11075657	10944671	276781	10451022
H8_3	11607901	149646	11334831	11184319	276338	10251135
H12_1	11372189	128586	11127213	11008713	313293	10129432
H12_2	11099975	155227	10778560	10559176	439778	9493752
H12_3	11118769	138865	10858748	10694699	291265	9859233

After a series of selections, a total of 303 miRNAs were identified, including 33 known miRNAs and 270 putative novel miRNAs (**Table 3**). The expression levels of miRNAs were calculated based on the read count and were subsequently normalized to TPM (number of transcripts per million clean tags). Among the 303 miRNAs, approximately 22% miRNAs were in a higher level (TPM interval > 60). However, approximately 25% miRNAs showed a much lower expression level (0 < TPM interval < 0.1) (**Supplementary Table 7**). Specifically, the TPM of novel miRNAs was mostly in a lower level, and the known miRNAs were mostly in a higher level (**Supplementary Table 8**). While comparison was performed between different groups, the overall expression pattern of miRNAs among the four groups was highly consistent (**Figure 4B**). Compared with the miRNA expression levels of uninfected group, 39 miRNAs showed significantly differential expression (*p* < 0.05), including 26, 7, and 6 DE–miRNAs in H2 vs H0, H8 vs H0, and H12 vs H0 comparisons, respectively (**Figure 4C**). Unsupervised hierarchical clustering revealed that all samples were clustered according to their respective groups, proving that the miRNA expression signatures were able to differentiate challenge groups from normal group (**Figure 4D**). Venn diagram revealed that some DE–miRNAs were differentially expressed at two or three comparisons (**Figure 4E**).

**Table 3:** Information of identified miRNAs.

Types	Known miRNAs				Novel miRNAs				
	Mapped mature	Mapped hairpin	Mapped uniq sRNA	Mapped total sRNA	Mapped mature	Mapped star	Mapped hairpin	Mapped uniq sRNA	Mapped total sRNA
Total	33	20	3954	9035607	270	185	283	16396	40095324
H0_1	32	20	331	787637	186	115	206	1346	3591558
H0_2	31	19	328	677689	189	119	209	1386	3015826
H0_3	31	19	327	837091	202	119	219	1435	3080677
H2_1	31	19	355	702417	194	122	217	1385	2960730
H2_2	32	19	334	752630	195	115	212	1326	3164662
H2_3	31	19	316	737257	183	118	209	1329	2880012
H8_1	32	19	324	759629	190	115	210	1346	3204006
H8_2	32	20	327	858333	195	118	217	1416	4636913
H8_3	32	19	320	703263	196	121	218	1362	3578285
H12_1	31	19	306	730537	195	111	213	1378	3373371
H12_2	32	19	342	732434	182	120	205	1377	3531040
H12_3	32	19	344	756690	197	122	212	1310	3078244

### Overview of mRNA Sequencing Data

To identify the expression levels of *P. olivaceus* mRNAs, 12 cDNA libraries (H0, H2, H8, and H12) were constructed and sequenced. After low-quality reads were filtered and sequences with Ns 287,208,722, 294,346,456, 254,332,520, and 329,139,946 were removed, clean reads were obtained from the H0, H2, H8, and H12 libraries, respectively. These data were deposited in NCBI database, with the BioProject number of PRJNA510440. Furthermore, 84.91%, 82.57%, 84.45%, and 84.83% of the clean reads from the H0, H2, H8, and H12 libraries were uniquely mapped to the *P. olivaceus* genome (**Table 4**).

**Table 4:** Information list of mRNA sequencing data.

Sample name	Raw reads	Clean reads	Clean bases	Error rate (%)	Q20 (%)	Q30 (%)	GC content (%)	Uniquely mapped
H0_1	91001552	85570680	12.84G	0.02	97.16	92.72	47.4	72705298
H0_2	98086488	92224408	13.83G	0.02	97.18	92.72	48.9	77964971
H0_3	1.16E+08	109413634	16.41G	0.02	96.99	92.32	48.85	93186834
H2_1	1.34E+08	125928236	18.89G	0.02	97.1	92.55	49.09	1.04E+08
H2_2	91713816	86756860	13.01G	0.02	97.19	92.72	49.33	71682126
H2_3	83268176	81661360	12.25G	0.02	97.29	92.87	49.59	67854043
H8_1	84134060	82485038	12.37G	0.02	97.33	92.97	49.47	69583688
H8_2	89841602	88149192	13.22G	0.02	97.33	92.98	49.26	74369462
H8_3	85281542	83698290	12.55G	0.01	97.39	93.1	49.37	70821118
H12_1	1.24E+08	121500194	18.23G	0.01	97.39	93.09	49.28	1.03E+08
H12_2	99739544	97782904	14.67G	0.02	97.33	92.96	49	82395474
H12_3	1.12E+08	109856848	16.48G	0.01	97.37	93.09	47.94	93450039

**Figure 5:**
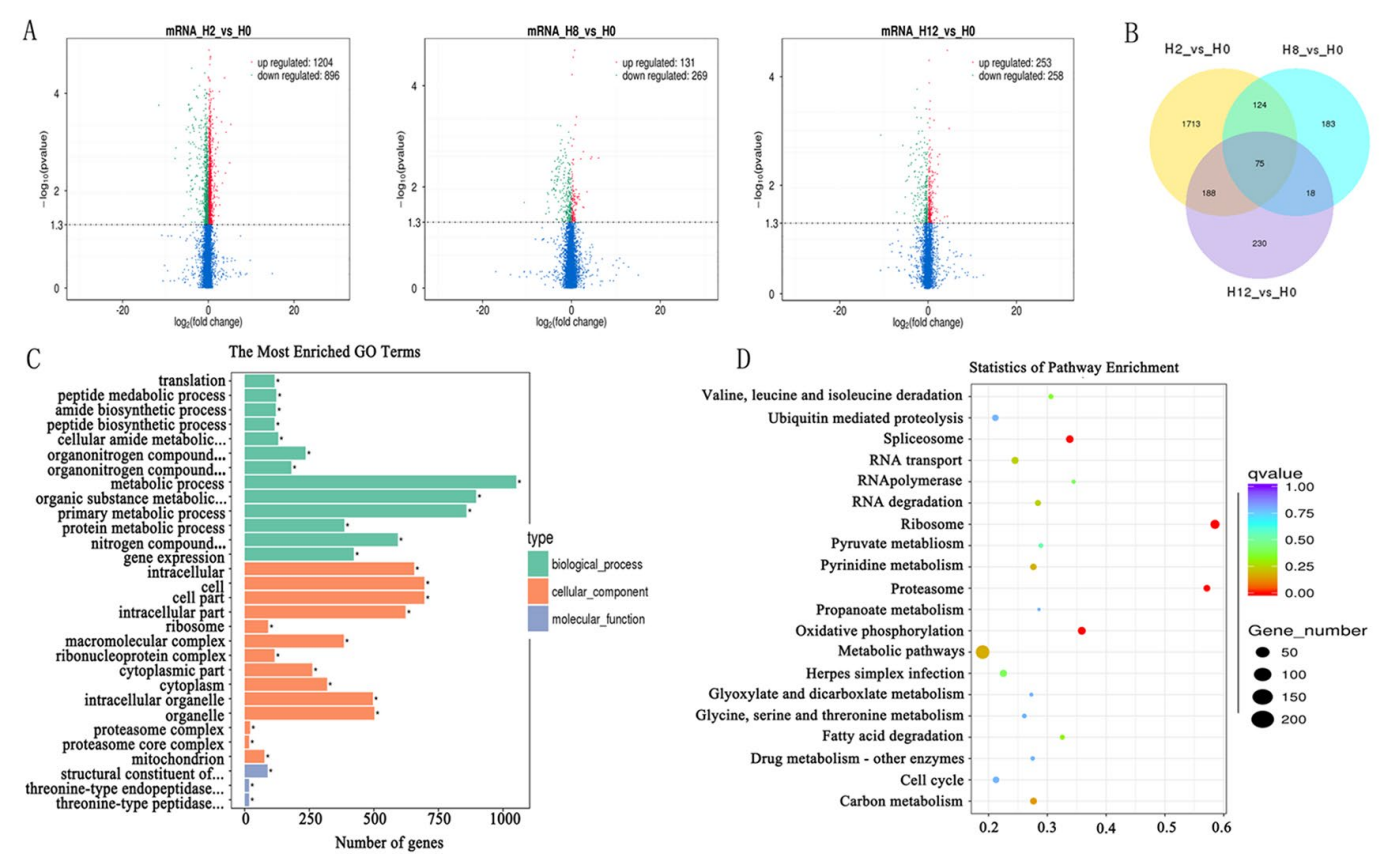
Analysis of mRNA sequencing data. Volcano plots for differential expression genes (DEGs) between H2, H8, H12, and H0, respectively. **(B)** Venn diagrams display the distribution of the 2,531 unique DEGs between H2, H8, H12, and H0, respectively. **(C)** Gene ontology (GO) analysis for all of DEGs between H2, H8, H12, and H0. *X*-axis represents the number of genes. The *Y*-axis on the left represents the GO term, and the *Y*-axis on the right represents GO type. The green column indicates the biological process, the red column the cellular component, and the gray column the molecular function. **(D)** Statistics of pathways enrichment of all DEGs between H2, H8, H12, and H0. Colors of the points refer to the *q*-value of the respective signaling pathway. Size of the point refers to the number of genes within each pathway.

In comparison with the H0 library, 2,100, 400, and 511 genes were identified as DEGs in H2, H8, and H12 libraries, respectively (**Figure 5A**, **Supplementary Table 9**). As shown in the Venn diagram, some DEGs were differentially expressed at two or three comparisons (**Figure 5B**). Considering that circRNAs could regulate transcription of their parental genes, we took the intersection of DE–circRNAs parental genes and DEGs. As shown in **Supplementary Table 9**, 13 DEGs served as parental genes of 13 DE–circRNAs. To further explore the functions of the DEGs in response to *E. tarda* infection, GO and KEGG enrichment analyses were conducted. For GO analysis, the dominant functions in each of the three main categories were metabolic process (GO:0008152) in the biological process (BP) category, cell (GO:0005623) in the cellular component (CC) category, and structural constituent of ribosome (GO:0003735) in the molecular function (MF) category (**Figure 5C**). In addition, the DEGs were aligned against the KEGG pathways database to identify pathways that were responsive to *E. tarda* infection. As shown in **Figure 5D**, the pathways of ribosome, proteasome, oxidative phosphorylation, and spliceosome were mostly activated.

### Construction of the Potential circRNA–miRNA Network

**Figure 6:**
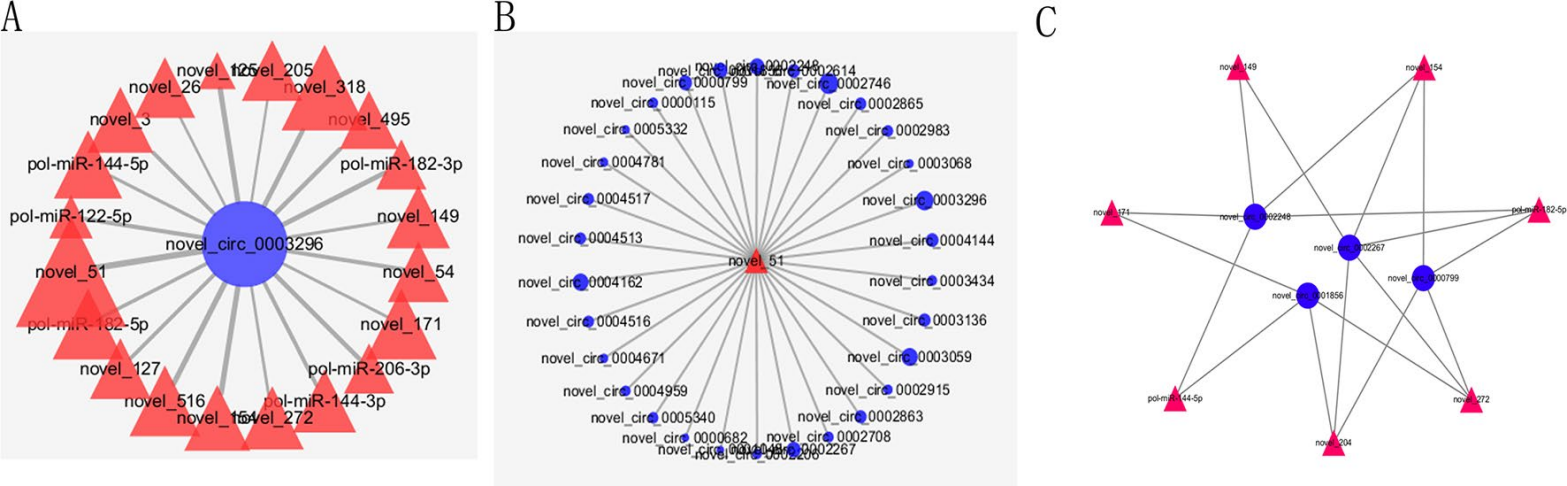
Partial circRNA–miRNA regulatory network. CircRNAs and miRNAs are indicated by blue circle and red triangle, respectively. **(A)** The miRNAs combined with novel_circ_0003296. **(B)** The circRNAs combined with novel_51. **(C)** The PPI network consisting of hub genes.

As we all know, circRNAs serving as competing endogenous RNA (ceRNA) of miRNA could regulate the expression of corresponding genes. In order to construct circRNA–miRNA regulation network, the MiRanda software was used to predict the relationship between DE–circRNAs and DE–miRNAs. The circRNA–miRNA network contained 325 circRNA–miRNA pairs, including 51 circRNAs and 32 miRNAs (**Supplementary Table 10**). As shown in **Figure 6A**, some circRNAs were predicted to combine several miRNAs; for example, novel_circ_0003296 could combine 20 miRNAs. Meanwhile, some miRNAs could also bind to several circRNAs; for example, novel_51 could link 30 circRNAs (**Figure 6B**). Considering the importance of hub genes in a network, we employed an MCODE approach (Bader and Hogue, 2003) to screen hub genes from the protein–protein interaction (PPI) network. With the k-core = 2, one subnetwork with 11 nodes and 18 edges was identified, including four circRNAs (novel_circ_0002248, novel_circ_0002267, novel_circ_0001856, and novel_circ_0000799) and seven miRNAs (novel_149, novel_154, novel_171, novel_204, novel_272, pol-miR-144-5p, and pol-miR-182-5p) (**Figure 6C**).

### Construction of the Potential miRNA–mRNA Network

In order to construct miRNA–mRNA regulation network, the MiRanda software was used to predict the relationship between DE–miRNAs and DEGs. We predicted the potential target genes of the 39 DE–miRNAs using MiRanda software. Then, we took the intersection of potential target genes and DEGs. We obtained in total 3,873 possible miRNA–mRNA target pairs under three comparisons. As shown in **Supplementary Table 11**, all miRNAs had more than one intersected DEGs. Five significant miRNAs, novel_51 (degree = 510), novel_144 (degree = 417), novel_171 (degree = 294), pol-miR-144-3p (degree = 218), and novel_318 (degree = 207), had the most target genes. Moreover, many mRNAs were associated with more than one miRNA, such as serpin H1 (gene_id, 109634651; transcript_id, XM_020095324.1), which was targeted by novel_265, novel_495, pol-miR-144-3p, novel_204, novel_17, novel_59, pol-miR-206-3p, novel_3, pol-let-7a-5p, novel_127, novel_154, and pol-miR-144-3p.

### Construction of the Potential circRNA–miRNA–mRNA Network

**Figure 7:**
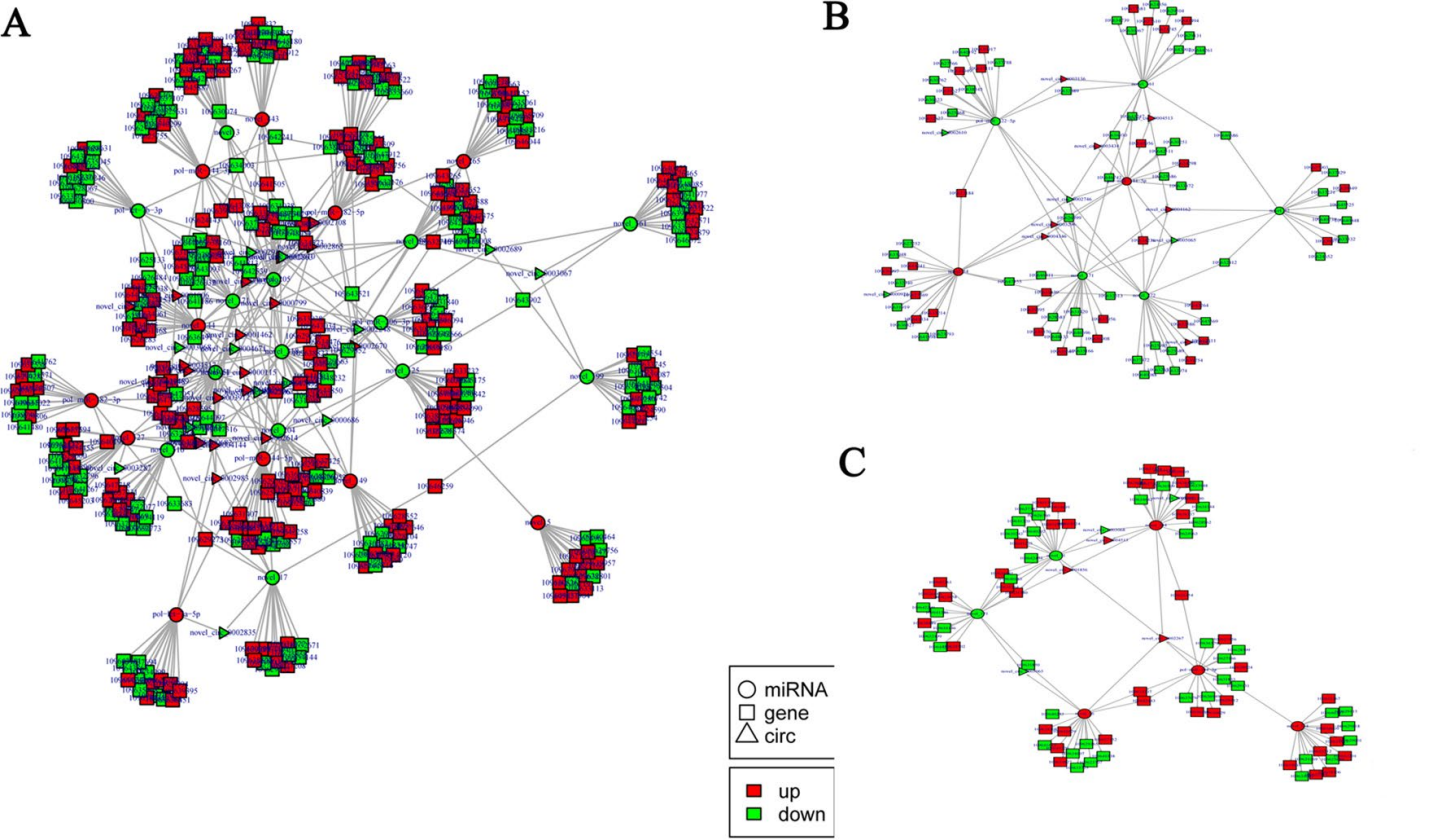
Functional circRNA–miRNA–mRNA regulatory modules between **(A)** H2, **(B)** H8, and **(C)** H12 and H0. CircRNA, miRNA, and mRNA are represented by triangles, circles, and squares, respectively. The red color represents up-regulated, and green color represents down-regulated.

According to the differentially expressed results, circRNA–miRNA pairs and miRNA–mRNA pairs were predicted by MiRanda software. Then, circRNA–miRNA–mRNA network was generated using a combination of data obtained from circRNA–miRNA pairs and miRNA–mRNA pairs (**Figure 7**). This network contained 198 circRNA–miRNA pairs and 3,873 miRNA–mRNA pairs, including 44 circRNAs, 32 miRNAs, and 1,774 mRNAs (**Supplementary Table 12**). Among 198 circRNA–miRNA pairs, four pairs of circRNA–miRNA existed in multiple comparison groups; for example, novel_circ_0005065-novel_171 existed in all three comparisons, novel_circ_0003068-novel_51 and novel_circ_0003068-novel_144 both existed in H2 vs H0 and H12 vs H0 comparisons, and novel_circ_0005065-pol-miR-144-5p existed in H2 vs H0 and H8 vs H0 comparisons. Among 3,873 miRNA–mRNA pairs, 178 miRNA–mRNA repeats existed in multiple comparison groups; for example, novel_171-109646742, novel_171-109646311, and novel_171-109644261 pairs existed in all of the three comparisons.

**Figure 8:**
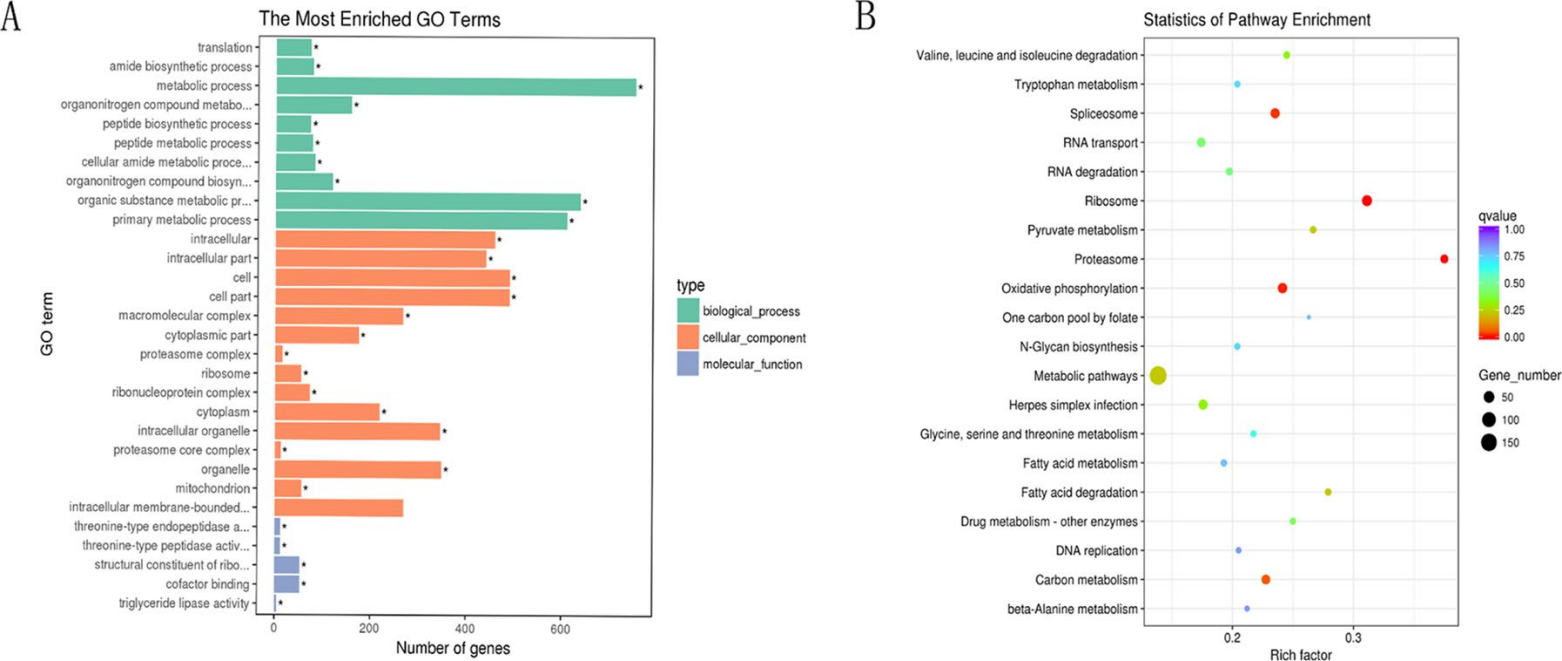
GO and KEGG analyses of the DEGs in the circRNA–miRNA–mRNA network. **(A)** GO analysis for DEGs in the circRNA–miRNA–mRNA network. *X*-axis represents the number of genes. The *Y*-axis on the left represents the GO term, and the *Y*-axis on the right represents GO type. The green column means the biological process, the red column means the cellular component, and the gray column means the molecular function. **(B)** Statistics of pathways enrichment of DEGs in the circRNA–miRNA–mRNA network. Color of the points refer to the *q*-value of the respective signaling pathway. Size of the point refers to the number of genes within each pathway.

Next, GO and KEGG analyses were performed to evaluate the function of the DEGs in the network. GO analysis revealed that there were 107, 53, and 96 enriched GO terms with statistical significance (*p* < 0.05) in the biological process, cellular component, and molecular function categories, respectively. GO analysis results suggested that some of the DEGs might play important biological roles during olive flounder against the *E. tarda* infection. As shown in **Figure 8A**, specific GO items were mainly involved in biological processes (e.g., metabolic process, organic substance metabolic process, and primary metabolic process), cell components (e.g., cell, cell part, and intracellular), and molecular function (e.g., catalytic activity, organic cyclic compound binding, and heterocyclic compound binding).

KEGG analysis revealed that there were 13 enriched terms with the statistical significance (*p* < 0.05), including metabolic pathways; ribosome; oxidative phosphorylation; spliceosome; herpes simplex infection; carbon metabolism; RNA transport; proteasome; RNA degradation; fatty acid degradation; pyruvate metabolism; valine, leucine, and isoleucine degradation; and drug metabolism (**Figure 8B**). Of those, herpes simplex infection pathway attracted considerable attention due to its involvement in immune response. A total of 32 DEGs are involved in herpes simplex infection pathway, including 109626283, 109623691, 109627599, 109644197, 109647155, 109641940, 109625845, 109637327, 109624406, 109633274, 109643961, 109636767, 109628267, 109643520, 109639858, 109644261, 109625570, 109634833, 109633363, 109643253, 109643252, 109637639, 109626354, 109631327, 109642261, 109641879, 109629246, 109641908, 109629344, 109646115, 109645569, and 109633948. With the exception of the above-mentioned herpes simplex infection pathway, some other immune-related pathways (*p* > 0.05) were also identified, for example, RIG-I-like receptor signaling pathway, *Salmonella* infection, apoptosis, intestinal immune network for IgA production, regulation of autophagy, toll-like receptor signaling pathway, endocytosis, phagosome, lysosome, and mitogen-activated protein kinase signaling pathway. Of those, intestinal immune network for IgA production (*p* < 0.05 in H8 vs H0 comparison) is a pathway, which is well known for its ability to generate great amounts of noninflammatory immunoglobulin A (IgA) antibodies that serve as the first line of defense against microorganisms. A total of three DEGs were involved in intestinal immune network for IgA production, including 109643253, 109643252, and 109633940.

### qRT-PCR Verification of Selected circRNAs, miRNAs, and mRNAs

**Figure 9:**
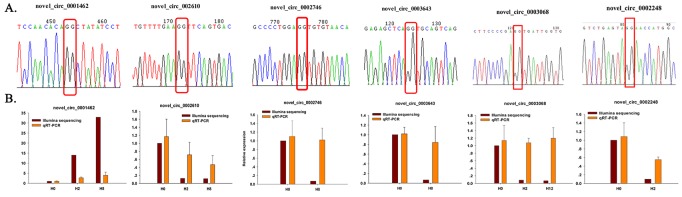
Confirmation of circRNAs by Sanger sequencing and qRT-PCR. **(A)** Sanger sequencing confirmation of the head-to-tail backsplicing of circRNAs. The nucleotides highlighted by red boxes indicate the junction sites. **(B)** qRT-PCR analysis result (orange) was compared with data obtained from Illumina sequencing (crimson). The relative expression level between H2, H8, H12, and H0 samples were showed by fold change. The data present the mean ± standard error (SE) derived from triplicate experiments. The relative expression levels by high-throughput sequencing analysis are represented by 2^log2(treatment/control)^.

To further confirm the expression level of circRNAs obtained by Illumina sequencing, six DE–circRNAs (novel_circ_0001462, novel_circ_0002610, novel_circ_0002746, novel_circ_0003643, novel_circ_0003068, and novel_circ_0002248) were selected for qRT-PCR. Divergent primers were designed for each selected circRNA, and EF1α was used as internal control (**Supplementary Table 13**). Specifically, divergent primers could only amplify circular RNA forms, but not genomic DNA or linearized mRNAs, and Sanger sequencing further confirmed the amplified products to be circRNAs (**Figure 9A**). Their relative expression level at different time points (H2, H8, and H12) was compared with that of H0. As shown in **Figure 9B**, most of the qRT-PCR results were consistent with those of Illumina sequencing. Therefore, the Sanger sequencing and qRT-PCR results confirmed the reliability and accuracy of the circRNA sequencing data.

**Figure 10:**
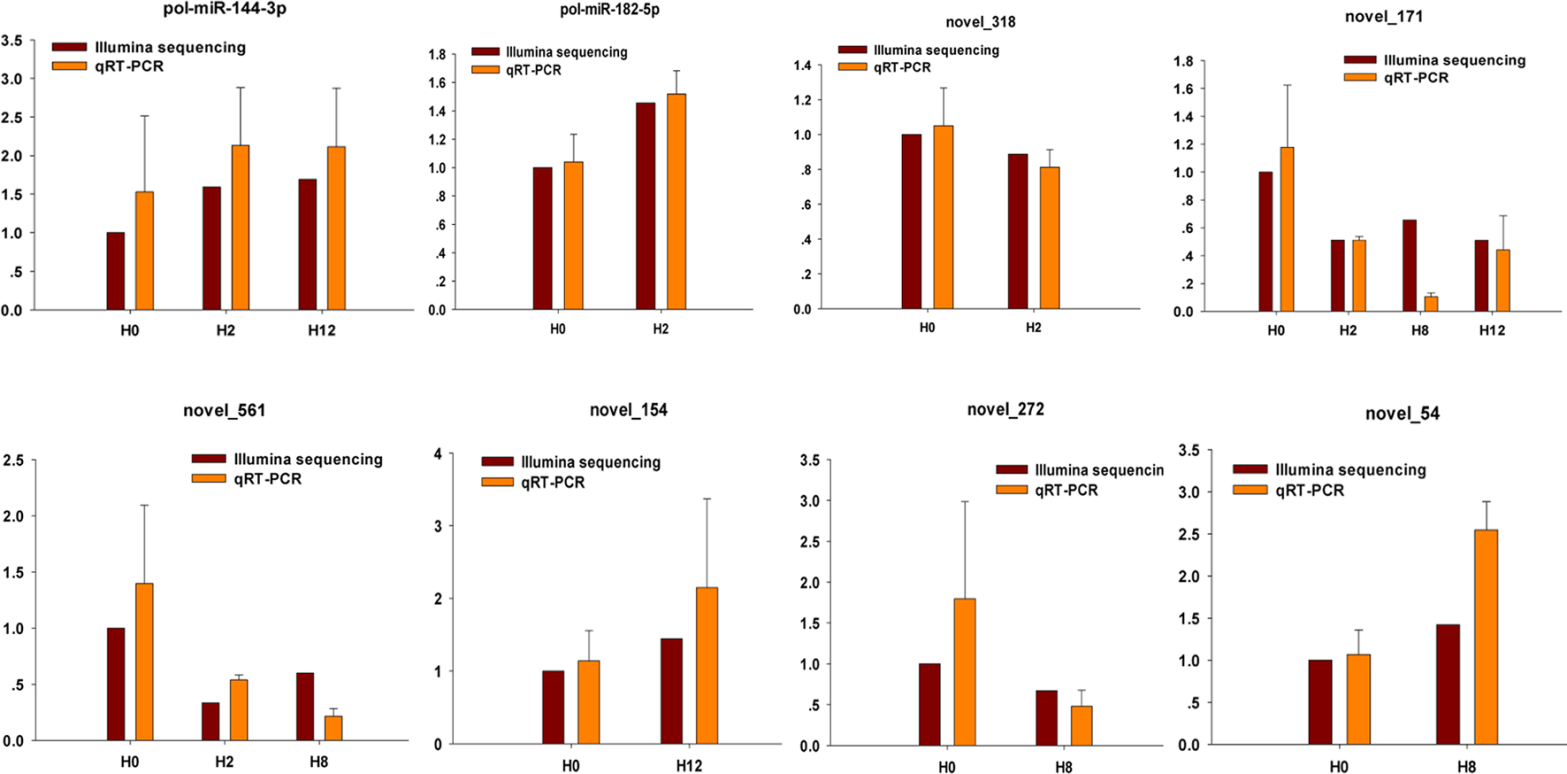
Confirmation of miRNAs by qRT-PCR analysis. qRT-PCR analysis result (orange) was compared with data obtained from Illumina sequencing (crimson). The expression rates of qRT-PCR between H2, H8, H12, and H0 samples are shown by fold change. The data present the mean ± standard error (SE) derived from triplicate experiments. The relative expression levels by high-throughput sequencing analysis are represented by 2^log2(treatment/control)^.

To further confirm the expression level of miRNAs obtained by Illumina sequencing, eight DE–miRNAs (pol-miR-144-3p, pol-miR-182-5p, novel_318, novel_171, novel_561, novel_154, novel_272, and novel_54) were selected for qRT-PCR. Different primers were designed for each selected miRNA, and 5S rRNA was used as internal control (**Supplementary Table 14**). As shown in **Figure 10**, there was similarity between the quantitative assay and high-throughput sequencing analysis of the eight miRNAs in terms of fold change and significance of differential expression. Although there were few differences in fold change of expression, the variation trend was identical.

**Figure 11:**
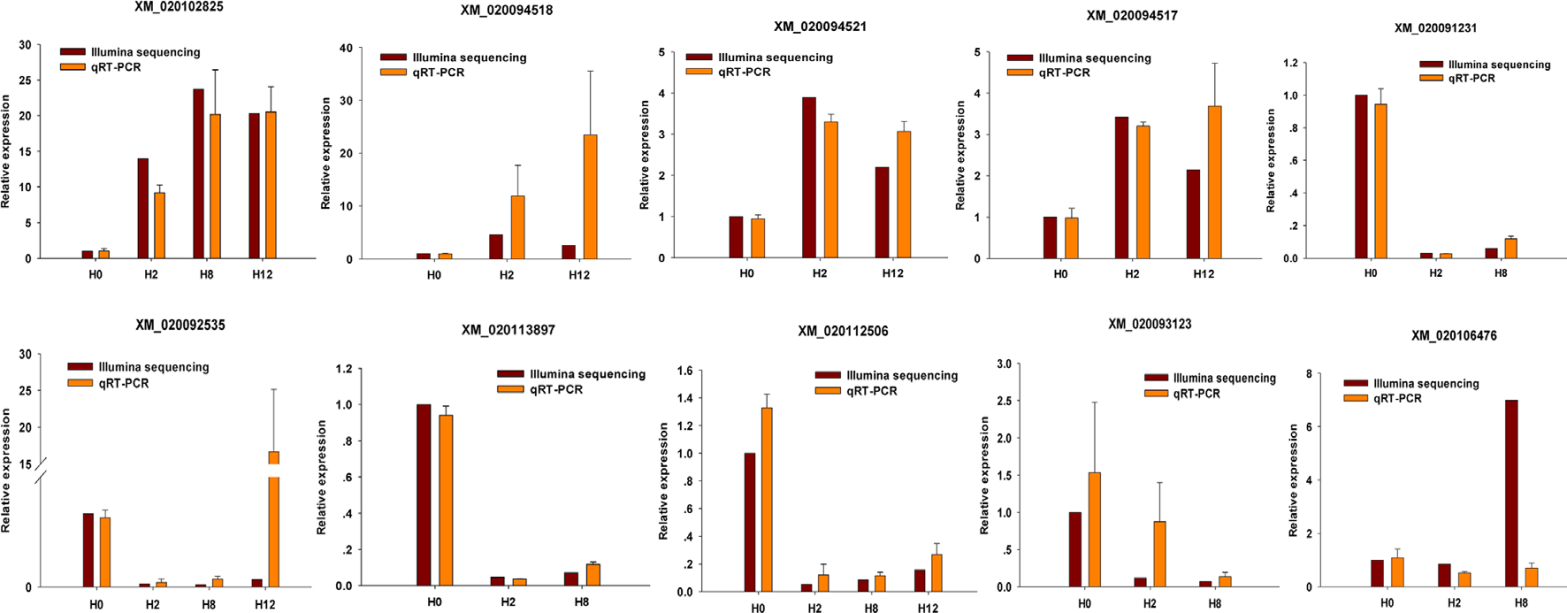
Confirmation of mRNAs by qRT-PCR analysis. qRT-PCR analysis result (orange) was compared with data obtained from Illumina sequencing (crimson). The relative expression level between H2, H8, H12, and H0 samples are shown by fold change. The data present the mean ± standard error (SE) derived from triplicate experiments. The relative expression levels by high-throughput sequencing analysis are represented by 2^log2(treatment/control)^.

To validate the expression patterns of the mRNAs, qRT-PCR was utilized to detect the expression of 10 randomly selected DEGs (XM_020102825, XM_020094518, XM_020094521, XM_020094517, XM_020091231, XM_020092535, XM_020113897, XM_020112506, XM_020093123, and XM_020106476). Different primers were designed for each selected mRNA, and EF1α was again used as internal control (**Supplementary Table 15**). As shown in **Figure 11**, all of 10 randomly selected DEGs showed a similar expression pattern between qRT-PCR and Illumina sequencing, although there were slight differences in the fold change.

## Discussion

Histopathological evaluation and cytokine expression analysis on the posterior intestine of *P. olivaceus* helped in understanding and determining the pathogenesis of *E. tarda*. This research described the morphological changes and cytokine expression changes of the posterior intestine from olive flounder after *E. tarda* infection at different time points. As the infection developed, the integrity of the intestinal mucosal structures showed pathological changes, such as cellular swelling, thickness of the lamina propria, shedding of the epithelial cells, and fragmentation of mucosal folds. Furthermore, structures of the posterior intestine, which were for immunity, especially the adaptive immunity, had seen an increased number of inflammatory cells (predominantly lymphocytes) and goblet cells at late infection (i.e., 8 and 12 h post *E. tarda* infection), which was consistent with qRT-PCR results of cytokine expression. Significantly thicker lamina propria, which was the structure for immunity, was observed at 8 and 12 h post-infection, an indication that the immune system actively participated at that particular time period. Pathogenesis study integrating morphological histology approach and next-generation sequencing approach would benefit a better understanding and elucidation of the intestinal immune response in the course of host–bacterial interaction.

More and more researches have proved that circRNAs, acting as miRNA sponges, counteract miRNA and eventually mediate expression of mRNAs (Hansen et al., 2013). Recently, the regulatory network of circRNA–miRNA–mRNA has been increasingly demonstrated in different kinds of diseases (Zheng et al., 2016; Chen et al., 2017a). In this research, we constructed a global circRNA–miRNA–mRNA network based on predicted circRNA–miRNA and miRNA–mRNA pairs in the pathogenesis of *E. tarda* in olive flounder. The integrated circRNA–miRNA–mRNA network consisted of 44 circRNAs, 32 miRNAs, and 1,774 mRNAs. GO and KEGG analyses were performed to evaluate the function of the DEGs in the network. KEGG analysis revealed that two important intestinal immune pathways (herpes simplex infection pathway and intestinal immune network for IgA production pathway) showed statistical significance between challenge and control groups.

Humans are the natural host of herpes simplex virus (HSV), of which HSV-1 resides in greater than 60% of the world’s population and causes peripheral disease (Farooq and Deepak, 2012). HSV-1 and HSV-2 initiate the infection process in epithelial cells of mucosal surfaces. Binding glycoproteins gB and gC of HSV-1 with heparan sulfate proteoglycans from host cell surface allows attachment of the viral glycoproteins gB, gD, and gL to host cellular receptors, such as nectin1, herpes virus entry mediator, or 3-*O*-sulfated HS for membrane fusion and viral entry (Agelidis and Shukla, 2015; Menendez and Carr, 2017). In addition, nectin2 also functions as a receptor for HSV-2, although the binding to the gD is weak. Consistent with these conclusions, CHO cell line expressing hNectin2 was susceptible to HSV-2 infection (Fujimoto et al., 2016). Coincidentally, we also found nectin2 (mRNA id, XM_020093024.1; gene id, 109633274) in the current circRNA–miRNA–mRNA network, and it was down-regulated between H2 and H0 comparison. Similar to HSV, the challenged fish were immersed in the bacteria solution so that their mucosal surfaces (gill, skin, and gastrointestinal tract) became important sites of bacterial exposure and colonization. Therefore, we concluded that nectin2 of *P. olivaceus* may be also a receptor of *E. tarda*, and the down-regulation of nectin2 is an active regulation of the host for self-protection. Then, we found several circRNA–miRNA–RNA networks where nectin2 is located, including 1) novel_circ_0002248/novel_circ_0002610/novel_circ_0003068/novel_circ_0004671-novel_144-nectin2 and 2) novel_circ_0000686/novel_circ_0002248/novel_circ_0005065-novel_149-nectin2. In the following study, we focused on the function of nectin2 and its circRNA–miRNA–mRNA regulatory network.

The intestine is one of the main mucosa-associated lymphoid tissues (MALT) of teleosts (Salinas, 2015). One striking feature of intestinal immunity is its ability to generate great amounts of noninflammatory immunoglobulin A (IgA), which would promote immune exclusion by entrapping dietary antigens and microorganisms in the mucus and function for neutralization of toxins and pathogenic microbes (Sidonia and Tasuku, 2003). In this research, three up-regulated DEGs were involved in intestinal immune network for IgA production pathway, including one MAP3K14 (mRNA id, XM_020094132.1; gene id, 109633940) and two components of MHC II (mRNA id, XM_020108298.1 and XM_020108299.1; gene id, 109643252 and 109643253). MHC II proteins present antigens to CD4^+^ T cells and interact with the T-cell receptor, followed by T-cell activation and cytokine secretion in immune responses (Wang et al., 1999; Bénichou and Benmerah, 2003). Besides, it was indicated that mice deficient in MHC class II expression (C2d mice) cannot produce IgA antibody whether protein antigens administered orally or against antigens from a protozoan parasite that colonized the small intestine (Snider et al., 1999). Therefore, we concluded that MHC II may play important roles in *P. olivaceus* against *E. tarda* infection. Then, we found several circRNA–miRNA–RNA networks where MHC II is located, including 1) novel_circ_0001006/novel_circ_0002863/novel_circ_0002865/novel_circ_0003059/novel_circ_0003296/novel_circ_0003912/novel_circ_0004144/novel_circ_0004162/novel_circ_0004346/novel_circ_0004959-novel_171-109643253, 2) novel_circ_0000115/novel_circ_0000682/novel_circ_0000799/novel_circ_0002614/novel_circ_0002708/novel_circ_0002863/novel_circ_0002865/novel_circ_0002983/novel_circ_0003059 novel_circ_0004144/novel_circ_0004516/novel_circ_0004517/novel_circ_0004959/novel_51-109643252, and 3) novel_circ_0000799/novel_circ_0002614/novel_circ_0003059/novel_circ_0003912-novel_205-109643252. The above-mentioned MHC II-associated regulatory network should be paid much more attention.

In conclusion, by employing Illumina sequencing, bioinformatics, and qRT-PCR technologies, we constructed circRNA–miRNA–mRNA networks and found two important intestinal immune pathways (herpes simplex infection pathway and intestinal immune network for IgA production pathway). In addition, three critical DEGs (nectin2, MHC II α-chain, and MHC II β-chain) were identified, and their circRNA–miRNA–mRNA networks were also constructed and discussed. Our study provides a novel insight into the immune response for *P. olivaceus* to *E. tarda* infection from the circRNA–miRNA–mRNA view. Future research on the specific mechanism of action should be investigated in the pathology of *E. tarda*.

## Author Contributions

YX: constructed the circRNA–miRNA–mRNA network and wrote this paper; GJ: raised *Paralichthys olivaceus* and conducted the bacteria challenge experiment; SZ, JD, and HL: analyzed the sequencing results of circRNA, miRNA, and mRNA; BS: performed the histopathological analysis on the intestine tissues and revised the manuscript; CL: conceived and designed the research, and revised the manuscript.

## Funding

This work was supported by Key Research and Invention program in Shandong Province (2017GHY215004); Key Research and Development Program of Shandong Province (2016GSF115026); the Open Fund of Shandong Key Laboratory of Disease Control in Mariculture (KF201804); Natural Science Foundation of Shandong Province (Grant No. ZR2019BC009); Advanced Talents Foundation of QAU (Grant No. 6651118016); Fish Innovation Team of Shandong Agriculture Research System (SDAIT-12-06); Major Agricultural Applied Technological Innovation Projects of Shandong Province; First-class Fishery Discipline programme in Shandong Province; Aquatic Animal Immunologic Agents Engineering Research Center of Shandong Province; and Graduate Innovation Program of Qingdao Agricultural University.

## Conflict of Interest Statement

The authors declare that the research was conducted in the absence of any commercial or financial relationships that could be construed as a potential conflict of interest.

## References

[B1] Agelidis A. M. Shukla D. (2015). Cell entry mechanisms of HSV: what we have learned in recent years. Future Virol. 10, 1145–1154. 10.2217/fvl.15.85 27066105 PMC4822157

[B2] Anne N. Gero D. Hakim T. Mark R. Nil Ratan S. Marco G. (2014). Atypical RNAs in the coelacanth transcriptome. J. Exp. Zool. B. Mol. Dev. Evol. 322, 342–351. 10.1002/jez.b.22542 24174405

[B3] Bénichou S. Benmerah A. (2003). The HIV nef and the Kaposi-sarcoma-associated virus K3/K5 proteins: “parasites” of the endocytosis pathway. Med. Sci. (Pairs) 19, 100-106. 10.1051/medsci/2003191100 12836198

[B4] Bader G. D. Hogue C. W. (2003). An automated method for finding molecular complexes in large protein interaction networks. BMC Bioinformatics 4, 2. 10.1186/1471-2105-4-2 12525261 PMC149346

[B5] Capel B. Swain A. Nicolis S. Hacker A. Walter M. Koopman P. (1993). Circular transcripts of the testis-determining gene Sry in adult mouse testis. Cell 73, 1019–1030. 10.1016/0092-8674(93)90279-Y 7684656

[B6] Chen L. L. (2016). The biogenesis and emerging roles of circular RNAs. Nat. Rev. Mol. Cell Biol. 17, 205-211. 10.1038/nrm.2015.32 26908011

[B7] Chen Y. Yuan B. Wu Z. Dong Y. Li Z. Zeng Z. (2017a). Microarray profiling of circular RNAs and the potential regulatory role of hsa_circ_0071410 in the activated human hepatic stellate cell induced by irradiation. Gene 629, 35–42. 10.1016/j.gene.2017.07.078 28774651

[B8] Chen Y. G. Kim M. V. Chen X. Batista P. J. Aoyama S. Wilusz J. E. (2017b). Sensing self and foreign circular RNAs by intron identity. Mol. Cell 67, 228–238. 10.1016/j.molcel.2017.05.022 28625551 PMC5610545

[B9] Conn S. Pillman K. Toubia J. Conn V. Salmanidis M. Phillips C. (2015). The RNA binding protein quaking regulates formation of circRNAs. Cell 160, 1125–1134. 10.1016/j.cell.2015.02.014 25768908

[B10] Enright A. J. John B. Gaul U. Tuschl T. Sander C. Marks D. S. (2003). MicroRNA targets in *Drosophila*. Genome Biol. 5, R1. 10.1186/gb-2003-5-1-r1 14709173 PMC395733

[B11] Errichelli L. Dini Modigliani S. Laneve P. Colantoni A. Legnini I. Capauto D. (2017). FUS affects circular RNA expression in murine embryonic stem cell-derived motor neurons. Nat. Commun. 8, 14741. 10.1038/ncomms14741 28358055 PMC5379105

[B12] Fan R. F. Cao C. Y. Chen M. H. Shi Q. X. Xu S. W. (2018). Gga-let-7f-3p promotes apoptosis in selenium deficiency-induced skeletal muscle by targeting selenoprotein K. Metallomics 10, 941–952. 10.1039/C8MT00083B 29905752

[B13] Farooq A. V. Deepak S. (2012). Herpes simplex epithelial and stromal keratitis: an epidemiologic update. Surv. Ophthalmol. 57, 448–462. 10.1016/j.survophthal.2012.01.005 22542912 PMC3652623

[B14] Friedländer M. R. Mackowiak S. D. Li N. Chen W. Rajewsky N. (2012). miRDeep2 accurately identifies known and hundreds of novel microRNA genes in seven animal clades. Nucleic Acids Res. 40, 37–52. 10.1093/nar/gkr688 21911355 PMC3245920

[B15] Fujimoto Y. Ozaki K. Iwamori N. Takakuwa H. Ono E. (2016). Accumulation of a soluble form of human nectin-2 is required for exerting the resistance against herpes simplex virus type 2 infection in transfected cells. Acta Virol. 60, 41–48. 10.4149/av_2016_01_41 26982466

[B16] Gao Y. Zhang J. Zhao F. (2018). Circular RNA identification based on multiple seed matching. Brief. Bioinform. 19, 803–810. 10.1093/bib/bbx014 28334140

[B17] Guarnerio J. Bezzi M. Jeong J. C. Paffenholz S. V. Berry K. Naldini M. M. (2016). Oncogenic role of fusion-circRNAs derived from cancer-associated chromosomal translocations. Cell 165, 289–302. 10.1016/j.cell.2016.03.020 27040497

[B18] Hansen T. B. Jensen T. I. Clausen B. H. Bramsen J. B. Finsen B. Damgaard C. K. (2013). Natural RNA circles function as efficient microRNA sponges. Nature 495, 384–388. 10.1038/nature11993 23446346

[B19] He L. Zhang A. Xiong L. Li Y. Huang R. Liao L. (2017). Deep circular RNA sequencing provides insights into the mechanism underlying grass carp reovirus infection. Int. J. Mol. Sci. 18, E1977. 10.3390/ijms18091977 28906455 PMC5618626

[B20] Hwang J. Y. Markkandan K. Kwon M. G. Seo J. S. Yoo S. I. Hwang S. D. (2018). Transcriptome analysis of olive flounder (*Paralichthys olivaceus*) head kidney infected with moderate and high virulent strains of infectious viral hemorrhagic septicaemia virus (VHSV). Fish Shellfish Immunol. 76, 293–304. 10.1016/j.fsi.2018.03.014 29530830

[B21] Hyeon Ho K. Yuki K. Subramanya S. Eun Kyung L. Jennifer L, M. Myriam G. (2009). HuR recruits let-7/RISC to repress c-Myc expression. Genes Dev. 23, 1743–1748. 10.1101/gad.1812509 19574298 PMC2720259

[B22] Jian H. Ma J. Wei L. Liu P. Zhang A. Yang B. (2018). Integrated mRNA, sRNA, and degradome sequencing reveal oilseed rape complex responses to *Sclerotinia sclerotiorum* (Lib.) infection. Sci. Rep. 8, 10987. 10.1038/s41598-018-29365-y 30030454 PMC6054686

[B23] Kanehisa M. Araki M. Goto S. Hattori M. Hirakawa M. Itoh M. (2008). KEGG for linking genomes to life and the environment. Nucleic Acids Res. 36, D480–D484. 10.1093/nar/gkm882 18077471 PMC2238879

[B24] Kim J. S. Harikrishnan R. Kim M. C. Balasundaram C. Heo M. S. (2010). Dietary administration of *Zooshikella* sp. enhance the innate immune response and disease resistance of *Paralichthys olivaceus* against *Streptococcus iniae*. Fish Shellfish Immunol. 29, 104–110. 10.1016/j.fsi.2010.02.022 20206273

[B25] Langmead B. Pop M. (2009). Ultrafast and memory-efficient alignment of short DNA sequences to the human genome. Genome Biol. 10, R25. 10.1186/gb-2009-10-3-r25 19261174 PMC2690996

[B26] Langmead B. Salzberg S. L. (2012). Fast gapped-read alignment with Bowtie 2. Nat. Methods 9, 357–359. 10.1038/nmeth.1923 22388286 PMC3322381

[B27] Lasda E. Parker R. (2014). Circular RNAs: diversity of form and function. RNA 20, 1829–1842. 10.1261/rna.047126.114 25404635 PMC4238349

[B28] Lauriano E. R. Pergolizzi S. Aragona M. Montalbano G. Guerrera M. C. Crupi R. (2019). Intestinal immunity of dogfish *Scyliorhinus canicula* spiral valve: a histochemical, immunohistochemical and confocal study. Fish Shellfish Immunol. 87, 490–498. 10.1016/j.fsi.2019.01.049 30711492

[B29] Li C. Zhang Y. Wang R. Lu J. Nandi S. Mohanty S. (2012). RNA-seq analysis of mucosal immune responses reveals signatures of intestinal barrier disruption and pathogen entry following *Edwardsiella ictaluri* infection in channel catfish, *Ictalurus punctatus*. Fish Shellfish Immunol. 32, 816–827. 10.1016/j.fsi.2012.02.004 22366064

[B30] Li J. Lv Y. Liu R. Yu Y. Shan C. Bian W. (2018a). Identification and characterization of a conservative W chromosome-linked circRNA in half-smooth tongue sole (*Cynoglossus semilaevis*) reveal its female-biased expression in immune organs. Fish Shellfish Immunol. 82, 531–535. 10.1016/j.fsi.2018.08.063 30176335

[B31] Li L. Guo J. Chen Y. Chang C. Xu C. (2017a). Comprehensive CircRNA expression profile and selection of key circRNAs during priming phase of rat liver regeneration. BMC Genomics 18, 80. 10.1186/s12864-016-3476-6 28086788 PMC5237265

[B32] Li X. Liu C. X. Xue W. Zhang Y. Jiang S. Yin Q. F. (2017b). Coordinated circRNA biogenesis and function with NF90/NF110 in viral infection. Mol. Cell 67, 214–227. 10.1016/j.molcel.2017.05.023 28625552

[B33] Li X. Yang L. Chen L. L. (2018b). The biogenesis, functions, and challenges of circular RNAs. Mol. Cell 71, 428–442. 10.1016/j.molcel.2018.06.034 30057200

[B34] Licata P. Tardugno R. Pergolizzi S. Capillo G. Aragona M. Colombo A. (2018). In vivo effects of PCB-126 and genistein on vitellogenin expression in zebrafish. Nat. Prod. Res. 2, 1–8. 10.1080/14786419.2018.1455048 29607746

[B35] Liu X. Chang X. Wu H. Xiao J. Gao Y. Zhang Y. (2014). Role of intestinal inflammation in predisposition of *Edwardsiella tarda* infection in zebrafish (*Danio rerio*). Fish Shellfish Immunol. 41, 271–278. 10.1016/j.fsi.2014.09.009 25224880

[B36] Lu X. Chen X. Mu M. Wang J. Wang X. Wang D. (2016). Genome-wide analysis of long noncoding RNAs and their responses to drought stress in cotton (*Gossypium hirsutum* L.). PloS One 11, e0156723. 10.1371/journal.pone.0156723 27294517 PMC4905672

[B37] Lukiw W. J. (2013). Circular RNA (circRNA) in Alzheimer’s disease (AD). Front. Genet. 4, 307. 10.3389/fgene.2013.00307 24427167 PMC3875874

[B38] Ma J. I. Kang S. Jeong H. B. Lee J. (2018). Isolation and expression analysis of stimulator of interferon gene from olive flounder, *Paralichthys olivaceus*. Fish Aquatic Sci. 21, 5. 10.1186/s41240-018-0083-2

[B39] Mao X. Cai T. Olyarchuk J. G. Wei L. (2005). Automated genome annotation and pathway identification using the KEGG Orthology (KO) as a controlled vocabulary. Bioinformatics 21, 3787–3793. 10.1093/bioinformatics/bti430 15817693

[B40] Memczak S. Jens M. Elefsinioti A. Torti F. Krueger J. Rybak A. (2013). Circular RNAs are a large class of animal RNAs with regulatory potency. Nature 495, 333–338. 10.1038/nature11928 23446348

[B41] Menendez C. M. Carr D. J. J. (2017). Defining nervous system susceptibility during acute and latent herpes simplex virus-1 infection. J. Neuroimmunol. 308, 43–49. 10.1016/j.jneuroim.2017.02.020 28302316 PMC5474347

[B42] Mohanty B. R. Sahoo P. K. (2007). Edwardsiellosis in fish: a brief review. J. Biosci. 32, 1331–1344. 10.1007/s12038-007-0143-8 18202458

[B43] Parra D. Korytar T. Takizawa F. Sunyer J. O. (2016). B cells and their role in the teleost gut. Dev. Comp. Immunol. 64, 150–166. 10.1016/j.dci.2016.03.013 26995768 PMC5125549

[B44] Pertea M. Kim D. Pertea G. M. Leek J. T. Salzberg S. L. (2016). Transcript-level expression analysis of RNA-seq experiments with HISAT, StringTie and Ballgown. Nat. Protoc. 11, 1650–1667. 10.1038/nprot.2016.095 27560171 PMC5032908

[B45] Pfaffl M. W. (2001). A new mathematical model for relative quantification in real-time RT-PCR. Nucleic Acids Res. 29, e45. 10.1093/nar/29.9.e45 11328886 PMC55695

[B46] Piwecka M. Glazar P. (2017). Loss of a mammalian circular RNA locus causes miRNA deregulation and affects brain function. Science 357, eaam8526. 10.1126/science.aam8526 28798046

[B47] Qu S. Yang X. Li X. Wang J. Gao Y. Shang R. (2015). Circular RNA: a new star of noncoding RNAs. Cancer Lett. 365, 141–148. 10.1016/j.canlet.2015.06.003 26052092

[B48] Rybak-Wolf A. Stottmeister C. Glažar P. Jens M. Pino N. Giusti S. (2015). Circular RNAs in the mammalian brain are highly abundant, conserved, and dynamically expressed. Mol. Cell 58, 870–885. 10.1016/j.molcel.2015.03.027 25921068

[B49] Salinas I. (2015). The mucosal immune system of teleost fish. Biology (Basel) 4, 525–539. 10.3390/biology4030525 26274978 PMC4588148

[B50] Sanger H. L. Klotz G. Riesner D. Gross H. J. Kleinschmidt A. K. (1976). Viroids are single-stranded covalently closed circular RNA molecules existing as highly base-paired rod-like structures. Proc. Natl. Acad. Sci. U S A 73, 3852–3856. 10.1073/pnas.73.11.3852 1069269 PMC431239

[B51] Shen Y. Guo X. Wang W. (2017). Identification and characterization of circular RNAs in zebrafish. FEBS Lett. 591, 213–220. 10.1002/1873-3468.12500 27878987

[B52] Sidonia F. Tasuku H. (2003). Intestinal IgA synthesis: regulation of front-line body defences. Nat. Rev. Immunol. 3, 63–72. 10.1038/nri982 12511876

[B53] Snider D. P. Liang H. Switzer I. Underdown B. J. (1999). IgA production in MHC class II-deficient mice is primarily a function of B-1a cells. Int. Immunol. 11, 191–198. 10.1093/intimm/11.2.191 10069417

[B54] Sonja P. Sabine M. (2015). RNA circularization strategies *in vivo* and *in vitro*. Nucleic Acids Res. 43, 2454–2465. 10.1093/nar/gkv045 25662225 PMC4344496

[B55] Su G. Morris J. H. Demchak B. Bader G. D. (2014). Biological network exploration with Cytoscape 3. Curr. Protoc. Bioinformatics 47, 81311–24. 10.1002/0471250953.bi0813s47 25199793 PMC4174321

[B56] Wang M. Yu F. Wu W. Zhang Y. Chang W. Ponnusamy M. (2017). Circular RNAs: a novel type of non-coding RNA and their potential implications in antiviral immunity. Int. J. Biol. Sci. 13, 1497–1506. 10.7150/ijbs.22531 29230098 PMC5723916

[B57] Wang R. F. Wang X. Atwood A. C. Topalian S. L. Rosenberg S. A. (1999). Cloning genes encoding MHC class II-restricted antigens: mutated CDC27 as a tumor antigen. Science 284, 1351–1354. 10.1126/science.284.5418.1351 10334988

[B58] Wang X. P. Yan M. C. Hu W. L. Chen S. B. Zhang S. L. Xie Q. L. (2012). Visualization of *Sparus macrocephalus* infection by GFP-labeled *Edwardsiella tardal*. Isr. J. Aquacult-Bamid 64.

[B59] Wen M. (2012). miREvo: an integrative microRNA evolutionary analysis platform for next-generation sequencing experiments. BMC Bioinformatics 13, 140. 10.1186/1471-2105-13-140 22720726 PMC3410788

[B60] Wilusz J. E. (2018). A 360° view of circular RNAs: from biogenesis to functions. Wiley Interdiscip. Rev. RNA 9, e1478. 10.1002/wrna.1478 29655315 PMC6002912

[B61] Wu R. Sheng X. Tang X. Xing J. Zhan W. (2018). Transcriptome analysis of flounder (*Paralichthys olivaceus*) gill in response to Lymphocystis disease virus (LCDV) infection: novel insights into fish defense mechanisms. Int. J. Mol. Sci. 19, E160. 10.3390/ijms19010160 29304016 PMC5796109

[B62] Xu S. Xiao S. Qiu C. Wang Z. (2017). Transcriptome-wide identification and functional investigation of circular RNA in the teleost large yellow croaker (*Larimichthys crocea*). Mar. Genomics 32, 71–78. 10.1016/j.margen.2016.12.004 28089131

[B63] Xu T. T. Zhang X. H. (2014). *Edwardsiella tarda*: an intriguing problem in aquaculture. Aquaculture 431, 129–135. 10.1016/j.aquaculture.2013.12.001

[B64] Young M. D. Wakefield M. J. Smyth G. K. Oshlack A. (2010). Gene ontology analysis for RNA-seq: accounting for selection bias. Genome Biol. 11, R14. 10.1186/gb-2010-11-2-r14 20132535 PMC2872874

[B65] Yu Y. Y. Kong W. (2018). Mucosal immunoglobulins protect the olfactory organ of teleost fish against parasitic infection. PloS Pathog. 14, e1007251. 10.1371/journal.ppat.1007251 30395648 PMC6237424

[B66] Zhang B. C. Zhang J. Sun L. (2014). In-depth profiling and analysis of host and viral microRNAs in Japanese flounder (*Paralichthys olivaceus*) infected with megalocytivirus reveal involvement of microRNAs in host-virus interaction in teleost fish. BMC Genomics 15, 1–15. 10.1186/1471-2164-15-878 25297525 PMC4200114

[B67] Zheng Q. Bao C. Guo W. Li S. Chen J. Chen B. (2016). Circular RNA profiling reveals an abundant circHIPK3 that regulates cell growth by sponging multiple miRNAs. Nat. Commun. 7, 11215. 10.1038/ncomms11215 27050392 PMC4823868

[B68] Zhong Y. Du Y. Yang X. Mo Y. Fan C. Xiong F. (2018). Circular RNAs function as ceRNAs to regulate and control human cancer progression. Mol. Cancer 17, 79. 10.1186/s12943-018-0827-8 29626935 PMC5889847

