# Supplementary material for: Identification of Potential Immune-Related circRNA–miRNA–mRNA Regulatory Network in Intestine of Paralichthys olivaceus During Edwardsiella tarda Infection
Source: Front Genet. 2019 Aug 14;10:731. doi: 10.3389/fgene.2019.00731 (PMC6702444; doi:10.3389/fgene.2019.00731)
Supplement: Supplementary file 1 [file Table_1.docx]

**Table S1.** The measurement of different structures in posterior intestine collected from *P. olivaceus* during *E. tarda* infection at H0 (control), H2, H8 and H12 time-points.

|  | H0 | | H2 | | H8 | | H12 | |
| --- | --- | --- | --- | --- | --- | --- | --- | --- |
| Structure | Range | Mean | Range | Mean | Range | Mean | Range | Mean |
| MF | 93-940 | 677±323^a^ | 50-514 | 296±159^b^ | 250-600 | 480±159^ab^ | 142-357 | 243±107^ab^ |
| LP | 21-36 | 29±6^a^ | 35-57 | 45±8^b^ | 57-85 | 71±10^c^ | 78-107 | 91±11^d^ |
| ICM | 40-93 | 64±20^a^ | 50-59 | 60±11^a^ | 64-100 | 77±14^a^ | 57-92 | 67±14^a^ |
| OLM | 40-66 | 56±10^a^ | 14-35 | 24±8^bc^ | 21-50 | 38±13^b^ | 14-21 | 19±4^c^ |

MF: height of mucosal folds; LP: thickness of lamina propria; ICM: thickness of inner circular muscular layer; OLM: thickness of outer longitudinal muscular layer. Data are presented in mean (µm) ± standard error of mean SEM from 24 collected samples. Structures in different samples were compared for morphological changes, the same superscript in lowercase letter within rows are not significantly different.
