# Supplementary material for: Identification of Potential Immune-Related circRNA–miRNA–mRNA Regulatory Network in Intestine of Paralichthys olivaceus During Edwardsiella tarda Infection
Source: Front Genet. 2019 Aug 14;10:731. doi: 10.3389/fgene.2019.00731 (PMC6702444; doi:10.3389/fgene.2019.00731)
Supplement: Supplementary file 2 [file Table_2.docx]

**Table S2.** qRT-PCR primers designed for each selected cytokine mRNA.

| **Primers** | **Sequences (5’-3’)** | | | **Amplification efficiency** |
| --- | --- | --- | --- | --- |
| Po-EF1α-qF | CATGGTCGTGACCTTCGCTC | | | 1.89 |
| Po-EF1α-qR | CTCGGGCATAGACTCGTGGT | | |  |
| PoIL-1β-qF | CGCTTCCCCAACTGGTACAT | | | 1.95 |
| PoIL-1β-qF | GTAGCACCGGGCATTCTTCT | | |  |
| PoIL-6-qF | CTCCAGTCGAATACGAGCCC | | | 1.99 |
| PoIL-6-qR | ACTCTTTCTGGTGGTGAGCG | | |  |
| PoIL-8-qF | CCCGTGGGTCATAAAGTGCT | | | 1.98 |
| PoIL-8-qR | TCCGCTTTAAGCTTCGGTGT | | |  |
| PoIL-10-qF | CAGGAGGCTCAGAGAGGACT | | | 1.95 |
| PoIL-10-qR | AGACTCCTCCACGCTCTGAT | | |  |
| PoIL-16-qF | TTCGACGAGCACAGAAGACC | | | 1.95 |
| PoIL-16-qR | TTGTGCATGTTGAGGCAACG | | |  |
| PoIL-17D-qF | GGGAAACACTCTCGATGGCA | | | 2.00 |
| PoIL-17D-qR | TGGAGGTCAGAGTCCTGTGT | | |  |
| PoTNFa-qF | GGGCTCAGTTTCTCGGCTTT | | | 1.89 |
| PoTNFa-qR | CCACACCAGCTTGTTTTCGG | | |  |
| PoG-CSF-qF | AGTGGACTAACGAACCTGCG | | | 1.89 |
| PoG-CSF-qR | CTCAGACTGCGGATCAGGTC | | |  |
|  | |  |  | |
