# Supplementary material for: Identification of Potential Immune-Related circRNA–miRNA–mRNA Regulatory Network in Intestine of Paralichthys olivaceus During Edwardsiella tarda Infection
Source: Front Genet. 2019 Aug 14;10:731. doi: 10.3389/fgene.2019.00731 (PMC6702444; doi:10.3389/fgene.2019.00731)
Supplement: Supplementary file 5 [file Table_5.docx]

**Table S5.** Information list of other non-coding RNAs.

| **Sample** | **rRNA** | **rRNA:+** | **rRNA:-** | **tRNA** | **tRNA:+** | **tRNA:-** | **snRNA** | **snRNA:+** | **snRNA:-** | **snoRNA** | **snoRNA:+** | **snoRNA:-** |
| --- | --- | --- | --- | --- | --- | --- | --- | --- | --- | --- | --- | --- |
| HO_1 | 15867 | 15843 | 24 | 4 | 4 | 0 | 4242 | 4238 | 4 | 5068 | 5062 | 6 |
| HO_2 | 10457 | 10443 | 14 | 2 | 2 | 0 | 2011 | 2006 | 5 | 3163 | 3156 | 7 |
| HO_3 | 12400 | 12395 | 5 | 6 | 6 | 0 | 1496 | 1495 | 1 | 1818 | 1811 | 7 |
| H2_1 | 14486 | 14476 | 10 | 7 | 7 | 0 | 3864 | 3856 | 8 | 2514 | 2506 | 8 |
| H2_2 | 14781 | 14769 | 12 | 4 | 4 | 0 | 3486 | 3479 | 7 | 2626 | 2623 | 3 |
| H2_3 | 10931 | 10918 | 13 | 3 | 2 | 1 | 2750 | 2744 | 6 | 3421 | 3417 | 4 |
| H8_1 | 25829 | 25826 | 3 | 1 | 1 | 0 | 3283 | 3280 | 3 | 2765 | 2758 | 7 |
| H8_2 | 9366 | 9351 | 15 | 1 | 1 | 0 | 2524 | 2523 | 1 | 4504 | 4503 | 1 |
| H8_3 | 8799 | 8790 | 9 | 4 | 4 | 0 | 1478 | 1474 | 4 | 1878 | 1876 | 2 |
| H12_1 | 8014 | 8007 | 7 | 4 | 4 | 0 | 1226 | 1221 | 5 | 2739 | 2729 | 10 |
| H12_2 | 10412 | 10400 | 12 | 7 | 7 | 0 | 1456 | 1451 | 5 | 2232 | 2225 | 7 |
| H12_3 | 7213 | 7203 | 10 | 4 | 3 | 1 | 2541 | 2537 | 4 | 2943 | 2942 | 1 |
