# Supplementary material for: Identification of Potential Immune-Related circRNA–miRNA–mRNA Regulatory Network in Intestine of Paralichthys olivaceus During Edwardsiella tarda Infection
Source: Front Genet. 2019 Aug 14;10:731. doi: 10.3389/fgene.2019.00731 (PMC6702444; doi:10.3389/fgene.2019.00731)
Supplement: Supplementary file 6 [file Table_6.docx]

**Table S6.** Information list of repeat sequences.

| **Sample** | **repeat** | **repeat:+** | **repeat:-** |
| --- | --- | --- | --- |
| HO_1 | 4973 | 4065 | 908 |
| HO_2 | 2131 | 1288 | 843 |
| HO_3 | 1613 | 905 | 708 |
| H2_1 | 2090 | 1256 | 834 |
| H2_2 | 1988 | 1204 | 784 |
| H2_3 | 2167 | 1389 | 778 |
| H8_1 | 2128 | 1205 | 923 |
| H8_2 | 2390 | 1495 | 895 |
| H8_3 | 1788 | 1042 | 746 |
| H12_1 | 1664 | 1000 | 664 |
| H12_2 | 2066 | 1258 | 808 |
| H12_3 | 1981 | 1304 | 677 |
