# Supplementary material for: Identification of Potential Immune-Related circRNA–miRNA–mRNA Regulatory Network in Intestine of Paralichthys olivaceus During Edwardsiella tarda Infection
Source: Front Genet. 2019 Aug 14;10:731. doi: 10.3389/fgene.2019.00731 (PMC6702444; doi:10.3389/fgene.2019.00731)
Supplement: Supplementary file 7 [file Table_7.docx]

**Table S7.** Statistics of miRNA expression level.

| **TPM Interval** | **0-0.1** | **0.1-0.3** | **0.3-3.57** | **3.57-15** | **15-60** | **>60** |
| --- | --- | --- | --- | --- | --- | --- |
| HO_1 | 76(25.08%) | 41(13.53%) | 60(19.80%) | 32(10.56%) | 24(7.92%) | 70(23.10%) |
| HO_2 | 90(29.70%) | 25(8.25%) | 65(21.45%) | 31(10.23%) | 26(8.58%) | 66(21.78%) |
| HO_3 | 75(24.75%) | 43(14.19%) | 61(20.13%) | 35(11.55%) | 22(7.26%) | 67(22.11%) |
| H2_1 | 78(25.74%) | 35(11.55%) | 63(20.79%) | 37(12.21%) | 22(7.26%) | 68(22.44%) |
| H2_2 | 76(25.08%) | 43(14.19%) | 59(19.47%) | 33(10.89%) | 24(7.92%) | 68(22.44%) |
| H2_3 | 89(29.37%) | 25(8.25%) | 63(20.79%) | 32(10.56%) | 26(8.58%) | 68(22.44%) |
| H8_1 | 81(26.73%) | 44(14.52%) | 51(16.83%) | 36(11.88%) | 24(7.92%) | 67(22.11%) |
| H8_2 | 76(25.08%) | 40(13.20%) | 65(21.45%) | 36(11.88%) | 22(7.26%) | 64(21.12%) |
| H8_3 | 75(24.75%) | 41(13.53%) | 68(22.44%) | 31(10.23%) | 23(7.59%) | 65(21.45%) |
| H12_1 | 86(28.38%) | 40(13.20%) | 59(19.47%) | 27(8.91%) | 26(8.58%) | 65(21.45%) |
| H12_2 | 82(27.06%) | 35(11.55%) | 59(19.47%) | 33(10.89%) | 25(8.25%) | 69(22.77%) |
| H12_3 | 69(22.77%) | 40(13.20%) | 62(20.46%) | 32(10.56%) | 28(9.24%) | 72(23.76%) |
