# Supplementary material for: Identification of Potential Immune-Related circRNA–miRNA–mRNA Regulatory Network in Intestine of Paralichthys olivaceus During Edwardsiella tarda Infection
Source: Front Genet. 2019 Aug 14;10:731. doi: 10.3389/fgene.2019.00731 (PMC6702444; doi:10.3389/fgene.2019.00731)
Supplement: Supplementary file 8 [file Table_8.docx]

**Table S8.** Information of identified miRNAs from Known miRNAs or Novel miRNAs.

|  | **Known miRNAs** | | | | | | **Novel miRNAs** | | | | | | |
| --- | --- | --- | --- | --- | --- | --- | --- | --- | --- | --- | --- | --- | --- |
| **TPM interval** | **> 60** | **15-60** | **3.57-15** | **0.3-3.57** | **0.1-0.3** | **0-0.1** | **> 60** | **15-60** | **3.57-15** | **0.3-3.57** | **0.1-0.3** | **0-0.1** |  |
| HO_1.tpm | 19 | 4 | 3 | 5 | 1 | 1 | 51 | 20 | 29 | 55 | 40 | 75 |  |
| HO_2.tpm | 18 | 5 | 3 | 5 | 0 | 2 | 48 | 21 | 28 | 60 | 25 | 88 |  |
| HO_3.tpm | 18 | 3 | 6 | 3 | 1 | 2 | 49 | 19 | 29 | 58 | 42 | 73 |  |
| H2_1.tpm | 18 | 4 | 5 | 4 | 0 | 2 | 50 | 18 | 32 | 59 | 35 | 76 |  |
| H2_2.tpm | 18 | 4 | 4 | 4 | 2 | 1 | 50 | 20 | 29 | 55 | 41 | 75 |  |
| H2_3.tpm | 18 | 4 | 4 | 4 | 1 | 2 | 50 | 22 | 28 | 59 | 24 | 87 |  |
| H8_1.tpm | 18 | 4 | 5 | 4 | 1 | 1 | 49 | 20 | 31 | 47 | 43 | 80 |  |
| H8_2.tpm | 18 | 4 | 4 | 4 | 2 | 1 | 46 | 18 | 32 | 61 | 38 | 75 |  |
| H8_3.tpm | 17 | 6 | 1 | 7 | 1 | 1 | 48 | 17 | 30 | 61 | 40 | 74 |  |
| H12_1.tpm | 18 | 5 | 0 | 8 | 0 | 2 | 47 | 21 | 27 | 51 | 40 | 84 |  |
| H12_2.tpm | 20 | 3 | 3 | 5 | 1 | 1 | 49 | 22 | 30 | 54 | 34 | 81 |  |
| H12_3.tpm | 21 | 3 | 5 | 2 | 1 | 1 | 51 | 25 | 27 | 60 | 39 | 68 |  |
