# Supplementary material for: Identification of Potential Immune-Related circRNA–miRNA–mRNA Regulatory Network in Intestine of Paralichthys olivaceus During Edwardsiella tarda Infection
Source: Front Genet. 2019 Aug 14;10:731. doi: 10.3389/fgene.2019.00731 (PMC6702444; doi:10.3389/fgene.2019.00731)
Supplement: Supplementary file 13 [file Table_13.docx]

**Table S13.** Divergent primers designed for each selected circRNA.

| **Primers** | **Sequences (5’-3’)** | | | **Amplification efficiency** | **R value** |
| --- | --- | --- | --- | --- | --- |
| Po-EF1α-qF | CATGGTCGTGACCTTCGCTC | | | 1.89 | ---- |
| Po-EF1α-qR | CTCGGGCATAGACTCGTGGT | | |  |  |
| Circ_0001462_Diver_qF | ACCTTCACCTTCCGTTCCAA | | | 1.86 | 0.981 |
| Circ_0001462_Diver_qR | CAAACTCATGGGCAGCTACC | | |  |  |
| Circ_0002610_Diver_qF | AAAAGCTAAGGGATGTGGTGT | | | 1.85 | 0.941 |
| Circ_0002610_Diver_qR | GGGAATGTGATCAACTGGACTG | | |  |  |
| novel_circ_0002746_Diver_qF | GCACACCTACCTGCCCCTGG | | | 1.81 | 1.000 |
| novel_circ_0002746_Diver_qR | GGCTGATCTCCTCTTGTCTGTCG | | |  |  |
| novel_circ_0003643_Diver_qF | TAGGCGACGGTGTGGAAGATC | | | 1.82 | 1.000 |
| novel_circ_0003643_Diver_qR | CCTCCTCTACGCCCTGACTGC | | |  |  |
| novel_circ_0003068_Diver_qF | TTTCTGGTCACTCGCTCACC | | | 1.88 | -0.001 |
| novel_circ_0003068_Diver_qR | CATTGTAGAGGTCACGTGCTG | | |  |  |
| novel_circ_0002248_Diver_qF | CCTCCCCCTCGGTACTTTCCA | | | 1.91 | 1.000 |
| novel_circ_0002248_Diver_qR | AGGTCCACACTGCGTCACAAT | | |  |  |
|  | |  |  | |  |
