# Supplementary material for: Identification of Potential Immune-Related circRNA–miRNA–mRNA Regulatory Network in Intestine of Paralichthys olivaceus During Edwardsiella tarda Infection
Source: Front Genet. 2019 Aug 14;10:731. doi: 10.3389/fgene.2019.00731 (PMC6702444; doi:10.3389/fgene.2019.00731)
Supplement: Supplementary file 14 [file Table_14.docx]

**Table S14.** qRT-PCR primers designed for each selected miRNA.

| **Primers** | **Sequences (5’-3’)** | **Amplification efficiency** | | | **R value** | |
| --- | --- | --- | --- | --- | --- | --- |
| Po5S rRNA-miF | GCTTACGGCCATACCACCCT | 1.81 | | | ---- | |
| pol-miR-144-3p-qF | GCGCGCTACAGTATAGATGATGTACTAT | 1.81 | | | 0.988 | |
| pol-miR-182-5p-qF | GCTTTGGCAATGGTAGAACTCACA | 1.82 | | | 1.000 | |
| novel_318-qF | GCTGAGGAGCTTTGACCAAGTCCA | 1.86 | | | 1.000 | |
| novel_171-qF | GCTTCACATGAACGACTCCTGAAG | 1.81 | | | 0.761 | |
| novel_561-qF | TGGACGGAGAACTGATAAGGGT | 1.83 | | | 0.781 | |
| novel_154-qF | TGGTGTTGTGAATCAGGCCG | 1.83 | | | 1.000 | |
| novel_272-qF | ATGCACCCCTCTGGAGAATGT | 1.88 | | | 1.000 | |
| novel_54-qF | GCTGCTCAGTAGGCAGTGTAGATC | 1.79 | | | 1.000 | |
|  | |  |  |  | |  |
