# Supplementary material for: Identification of Potential Immune-Related circRNA–miRNA–mRNA Regulatory Network in Intestine of Paralichthys olivaceus During Edwardsiella tarda Infection
Source: Front Genet. 2019 Aug 14;10:731. doi: 10.3389/fgene.2019.00731 (PMC6702444; doi:10.3389/fgene.2019.00731)
Supplement: Supplementary file 15 [file Table_15.docx]

**Table S15.** qRT-PCR primers designed for each selected mRNA.

| **Primers** | **Sequences (5’-3’)** | **Amplification efficiency** | | | **R value** |
| --- | --- | --- | --- | --- | --- |
| Po-EF1α-qF | CATGGTCGTGACCTTCGCTC | 1.89 | | | ---- |
| Po-EF1α-qR | CTCGGGCATAGACTCGTGGT |  |  |  |  |
| XM_020102825-qF | GACGTCACCAAGGTCGTGTC | 2.09 | | | 0.968 |
| XM_020102825-qR | GGACAACCGATGTGTAACAG |  |  |  |  |
| XM_020094518-qF | GCATGGCAAGACAGTGATGG | 2.23 | | | 0.413 |
| XM_020094518-qR | AACCACAGCCAGGAACTTCT |  |  |  |  |
| XM_020094521-qF | GAAGGTCGGTGAGATCGGAG | 1.91 | | | 0.860 |
| XM_020094521-qR | TTGTAGGCATGCAGCTCACT |  |  |  |  |
| XM_020094517-qF | ACTCTGAGAAGCTCCATGTG | 1.96 | | | 0.749 |
| XM_020094517-qR | CTGAGCCTCAGTGGTACTGT |  |  |  |  |
| XM_020091231-qF | GACATTCAGTGCTGCAGTCA | 1.94 | | | 0.998 |
| XM_020091231-qR | ACGTCACGCTTCTGTCTGTT |  |  |  |  |
| XM_020092535-qF | GCAGGAGCTGGTTGACAAGT | 1.81 | | | -0.222 |
| XM_020092535-qR | AAGCCACTGGCAATCTTAGC |  |  |  |  |
| XM_020113897-qF | GACGCCTGAAGAATGTTCAC | 1.87 | | | 0.998 |
| XM_020113897-qR | CGTTAGCTACCGAGCTGATG |  |  |  |  |
| XM_020112506-qF | AGCCAGTTCACCATCCAATC | 1.85 | | | 0.999 |
| XM_020112506-qR | GGAAGCCAGTTGTCGTTGTC |  |  |  |  |
| XM_020093123-qF | GGTACGATGTTCCCTGCTCC | 1.95 | | | 0.871 |
| XM_020093123-qR | GGAGATGGGGGTGAAGGTTG |  |  |  |  |
| XM_020106476-qF | CCCGACGACCTTGTTTGAGA | 1.94 | | | -0.192 |
| XM_020106476-qR | CCAGTTGTGGTCTCGTCCTC |  |  |  |  |
|  | | |  |  |  |
